# Familial analysis reveals rare risk variants for migraine in regulatory regions

**DOI:** 10.1007/s10048-020-00606-5

**Published:** 2020-02-19

**Authors:** Tanya Ramdal Techlo, Andreas Høiberg Rasmussen, Peter L. Møller, Morten Bøttcher, Simon Winther, Olafur B. Davidsson, Isa A. Olofsson, Mona Ameri Chalmer, Lisette J. A. Kogelman, Mette Nyegaard, Jes Olesen, Thomas Folkmann Hansen

**Affiliations:** 1 Danish Headache Center, Department of Neurology, Rigshospitalet, Nordstjernevej 40, DK-2600 Glostrup, Denmark; 2 Department of Biomedicine, Aarhus University, Hoegh-Guldbergs Gade 10, Aarhus, Denmark; 3 Department of Cardiology, Hospital Unit West Jutland, Herning, Denmark; 4 Department of Cardiology, Aarhus University Hospital, Skejby, Aarhus, Denmark; 5 Institute for Biological Psychiatry, Mental Health Center Sct. Hans, Roskilde, Denmark; 6 Novo Nordic Foundation Centre for Protein Research, Copenhagen University, Copenhagen, Denmark

**Keywords:** Genetics, Genome-wide, Rare-variant association analysis, Migraine, Gene regulation, Family study

## Abstract

The most recent genome-wide association study of migraine increased the total number of known migraine risk loci to 38. Still, most of the heritability of migraine remains unexplained, and it has been suggested that rare gene dysregulatory variants play an important role in migraine etiology. Addressing the missing heritability of migraine, we aim to fine-map signals from the known migraine risk loci to regulatory mechanisms and associate these to downstream genic targets. We analyzed a large cohort of whole-genome sequenced patients from extended migraine pedigrees (1040 individuals from 155 families). We test for association between rare variants segregating in regulatory regions with migraine. The findings were replicated in an independent case-control cohort (2027 migraineurs, 1650 controls). We report an increased burden of rare variants in one CpG island and three polycomb group response elements near four migraine risk loci. We found that the association is independent of the common risk variants in the loci. The regulatory regions are suggested to affect different genes than those originally tagged by the index SNPs of the migraine loci. Families with familial clustering of migraine have an increased burden of rare variants in regulatory regions near known migraine risk loci, with effects that are independent of the variants in the loci. The possible regulatory targets suggest different genes than those originally tagged by the index SNPs of the migraine loci.

**Electronic supplementary material:**

The online version of this article (10.1007/s10048-020-00606-5) contains supplementary material, which is available to authorized users.

## Introduction

Migraine is a debilitating and complex genetic disorder with 1 billion affected individuals worldwide and a life-time prevalence of 15–20% [1]. Given the high prevalence, migraine has a considerable social and economic impact, with annual expenses estimated at €27 billion in Europe alone [2]. Furthermore, the Global Burden of Disease Study (2016) ranks migraine the second highest cause of disability worldwide [3]. Migraine is characterized by episodes of moderate to severe throbbing, unilateral headache, which intensifies with an increase in physical activity, and is accompanied by nausea and increased sensitivity to light and sound [4]. Rare variants with large effect sizes may be implicated with a high migraine risk [5]. The most recent meta-analysis on migraine genome-wide association studies (GWAS) identified 44 independent SNPs defining 38 migraine risk loci [6]. However, a GWAS typically assesses common SNPs derived from imputation and genotyping, and thus effect sizes are small. Using next generation sequencing, it is possible to assess rare variants. Such rare variants are expected to have a larger effect size, particularly if the variant segregates with disease within families [7]. Common complex disorders, such as migraine, are thought to be influenced by alterations in gene regulation [8, 9]. As migraine symptoms occur episodically and can be affected by regulatory factors, such as hormones [10, 11], it is very likely that gene dysregulation plays an important role in migraine etiology. SNPs that affect gene regulation or are present in regulatory regions have been linked with migraine [12, 13], which further supports the impact of regulatory dysfunction on the disorder. However, most of these SNPs are common with small effect sizes and fail to explain a high migraine risk.

We assessed whether rare variants in regulatory regions in known migraine risk loci have an increased risk burden for migraine. Additionally, we investigated whether the rare regulatory variants had effects that are independent of the variants in the migraine risk loci. We employed a cohort of extended families with familial clustering of migraine and performed whole-genome sequencing to identify rare variants. The findings were replicated in an independent case-control cohort of sporadic migraineurs with no familial history of migraine and controls.

## Materials and methods

### Familial cohort

A total of 155 families with familial clustering of migraine were recruited. The families consisted of 1040 subjects (mean number of individuals per family = 6.7) of which 746 individuals were diagnosed with migraine (mean number of migraineurs per family = 4.8) and 294 had no history of migraine. The smallest families consisted of at least two individuals and at least one migraineur. A proband from each family was initially recruited at the Danish Headache Center, Rigshospitalet-Glostrup, Denmark, and the remaining family members were subsequently recruited, as described elsewhere [14]. All subjects were assessed by a neurologist or a senior medical student trained in headache diagnostics using a validated semi-structured interview [15, 16] based on the International Classification of Headache Disorders [4].

### Replication cohort

The replication cohort consisted of 2027 sporadic migraineurs (no first-degree relatives with migraine) and 1650 controls. The sporadic migraineurs were recruited and interviewed at the Danish Headache Center using the same procedures as described for the probands of the familial cohort. The controls were recruited for the Danish study of Non-Invasive testing in Coronary Artery Disease (Dan-NICAD) according to procedures described by Nissen et al. [17].

### Sequencing

Genomic DNA was extracted from whole blood. All samples from both the familial cohort and the replication cohort were subjected to the same whole-genome sequencing procedures using an Illumina NovaSeq 6000 sequencing platform and S4 flow cells and subsequently subjected to quality control by deCODE genetics, as described elsewhere [18].

### Defining genomic regions for analysis

All regulatory regions surrounding the index SNP defining the known, autosomal migraine risk loci [6] were analyzed. We defined the genomic search regions as 1 Mb pairs upstream and downstream of the index SNP (see supplementary Table 1 for regions). In the 2 Mb pair window, the genomic positions (according to the Genome Reference Consortium Human Build 38 GRCh38.p12 (hg38)) of all regulatory regions were annotated using BED files downloaded from the UCSC Table browser [19]. These BED files included insulators [20, 21], polycomb group response elements (PREs) [20, 21], enhancers [22], transcription factor binding sites (TFBS) [21], CpG islands [23], and promoters [24]. All BED files were downloaded on 02/21/2019 except for the BED file of the PREs (downloaded on 02/28/2019). Any genomic coordinates from the hg19 assembly were subsequently converted to the hg38 assembly. In total, 1079 insulators; 518 PREs; 1651 enhancers; 16,190 TFBSs; 828 CpG islands; and 135 promoters were mapped and subjected to familial association analysis. Within the regulatory regions, only genetic variants with a minor allele frequency (MAF) < 5% were assessed.

### Familial association analysis

VCF files of each participant in the study were merged, multiallelic sites were split into a biallelic representation, the positions of the regulatory regions were annotated, and rare genetic variants (MAF < 5%) were isolated. The rare-variant association analysis was carried out with the software of Family Sequence Kernel Association Test (F-SKAT) [25]. Age and gender were used as covariates. The age was defined as the age at the time of the interview (the time of diagnosis). The analysis was performed with all default options in effect, and the resulting *p* values were controlled for multiple testing using the Bonferroni method. A Bonferroni-corrected *p* value *<* 0.05 was considered significant. To assess whether the findings were dependent on the original index SNP of the migraine GWAS, the number of alleles of the index SNP (0, 1, or 2) were applied as additional covariates in a separate rare-variant association analysis with F-SKAT.

### Replication

The findings from the familial association analysis were assessed in a case-control design using an independent cohort of sporadic migraineurs with no familial history of migraine and controls. Aggregated data were available on rare variants (MAF < 5%) in the regulatory regions for the controls. Thus, we tested whether the frequency of rare variants was increased in sporadic migraineurs compared to the controls under the null hypothesis of no association with migraine and under a dominant model of penetrance, using a χ^2^ test [26]. The resulting *p* values were controlled for multiple testing using the Bonferroni method. A Bonferroni-corrected *p* value *<* 0.05 was considered significant.

### eQTL and gene expression analysis

Regulatory regions with an increased burden of rare variants were subsequently assessed using gene expression data and single-tissue *cis*-eQTL data from the Genotype-Tissue Expression (GTEx) version 8.0 dataset (dbGaP Accession phs000424.v8.p2, 2019-08-26 release). All SNP identifiers were found in the Database of Single Nucleotide Polymorphisms (dbSNP) [27] build 152.

## Results

### Increased burden of rare variants found in four regulatory regions

We analyzed 155 migraine families (*n* = 1040) and 37 autosomal migraine risk loci (supplementary Table 1) and found a significant increased burden of rare variants segregating with migraine in 39 regulatory regions (see supplementary Table 2 for regulatory regions and *p* values). As expected, when analyzing rare variants in regions, that are known to harbor polygenic effects in the known risk loci [6, 28, 29], we found that the distribution of the test statistics deviated from the null, with a genomic inflation factor of λ = 1.89 (supplementary Fig. 1).

**Fig. 1:**
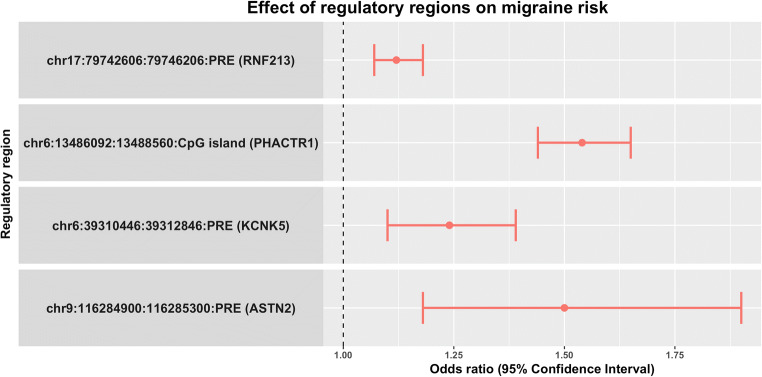
Effect of regulatory regions on migraine risk. The figure presents the effect of the significant regulatory regions on migraine risk. X-axis: OR (dot) with the 95% confidence intervals (lines). Y-axis: The genomic positions (given as chromosome:start:end) and type of regulatory regions and the migraine risk loci in brackets

In an independent case-control cohort using sporadic migraineurs with no familial history of migraine (*n*_*cases*_ = 2027) and controls (*n*_*controls*_ = 1650), assuming a dominant model of penetrance, we verified that four of the regulatory regions had an increased burden of rare variants (Table 1). The four regulatory regions include a CpG island located near the *PHACTR1* migraine risk locus and three PREs located near the *KCNK5*, *ASTN2*, and *RNF213* migraine risk locus. The CpG island located near the *PHACTR1* migraine risk locus associated with the highest migraine risk, having an odds ratio (OR) of 1.54 (Fig. 1). Extensive information of the rare variants identified for the four regulatory regions is available in supplementary Table 3.

**Table 1:** Results from replication using an independent case-control cohort of sporadic migraineurs and controls. The table presents the name and MAF of the index SNP of the migraine risk loci, as given by Gormley et al. (2016) [6], the genomic positions of the regulatory regions, the type of regulatory regions, the Bonferroni-corrected *p* values, and the ORs with the 95% confidence intervals (CI)

Locus	MAF	Position (chromosome:start:end)	Type	*p* value	OR (95% CI)
*PHACTR1*	0.41	chr6:13486092:13488560	CpG island	5.3·10^−15^	1.54 (1.44–1.65)
*KCNK5*	0.28	chr6:39310446:39312846	PRE	0.014	1.24 (1.1–1.39)
*ASTN2*	0.36	chr9:116284900:116285300	PRE	0.017	1.5 (1.18–1.9)
*RNF213*	0.17	chr17:79742606:79746206	PRE	9.3·10^−5^	1.12 (1.07–1.18)

### Association of migraine and rare regulatory variants independent of known risk loci

Subsequently, we tested whether the four regulatory regions with increased burden were correlated with the index SNP of the migraine risk loci. Using the risk allele count of the index SNP as covariate, we found a nonsignificant effect on the results defining each migraine risk locus as additional covariates (Table 2).

**Table 2:** Results obtained with familial association analysis using the presence of the index SNP as additional covariates. The table gives the name of the migraine risk loci, the type of regulatory regions, and the Bonferroni-corrected *p* values with and without applying the presence of the index SNP as additional covariates

Locus	Type	*p* value without allele count of index SNP as covariate	*p* value with allele count of index SNP as covariate
*PHACTR1*	CpG island	0.044	0.042
*KCNK5*	PRE	0.011	0.012
*ASTN2*	PRE	0.011	0.011
*RNF213*	PRE	5.7·10^−3^	5.8·10^−3^

### Possible regulatory targets identified for three regulatory regions

**Fig. 2:**
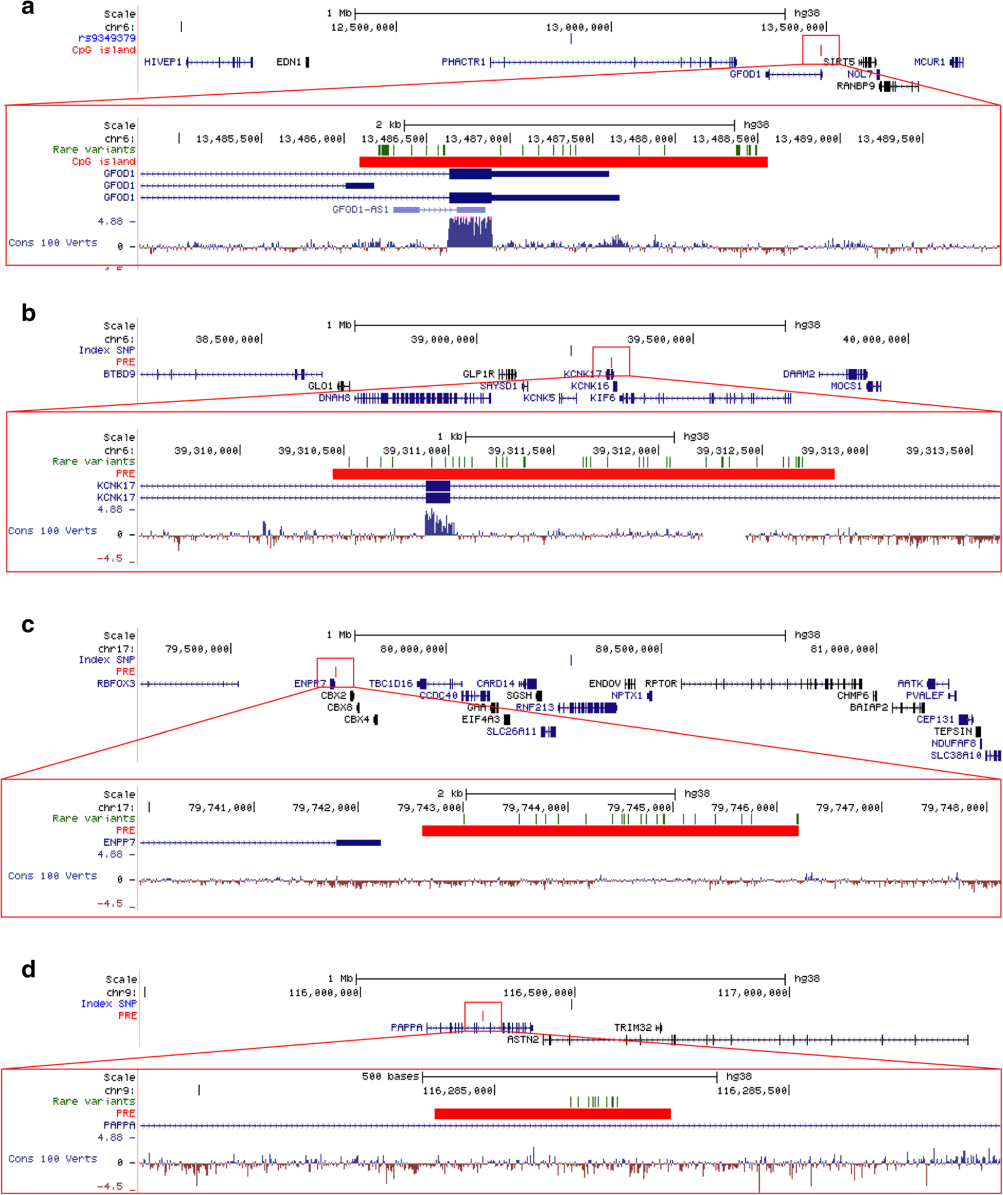
The genomic regions in which the four regulatory regions are located. In the top of each figure is 1 Mb pairs upstream and downstream of the index SNP defining the migraine risk loci displayed. Within this 2 Mb pair window, regulatory regions were annotated and rare variants within these were assessed. The index SNP defining the migraine risk loci is located in the middle, and below are the regulatory regions (the CpG island and the PREs) in which rare variants associated with an increased migraine risk and genic transcript information. In the bottom of each figure is a close-up of the regulatory regions. Here, the rare variants, that had an increased burden in the familial association analysis, are indicated. The genomic regions surrounding **a** the *PHACTR1* locus and the CpG island, **b** the *KCNK5* locus and the PRE, **c** the *RNF213* locus and the PRE, and **d** the *ASTN2* locus and the PRE

**Fig. 3:**
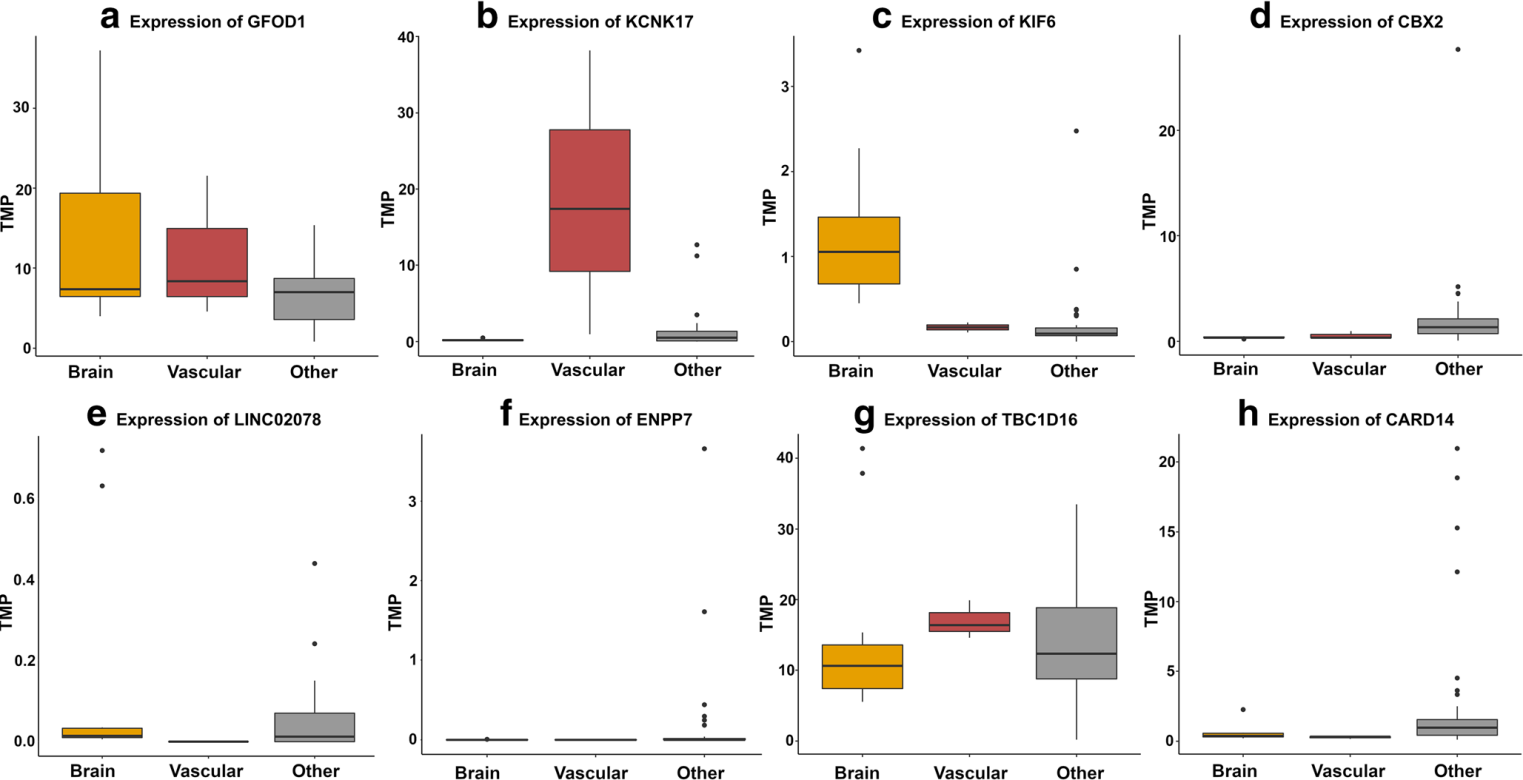
Expression of the possible genic regulatory targets across different tissues. The figure displays expression data from the GTEx database where the tissue types have been divided according to tissue group (brain, vascular, and other remaining tissue). Y-axis: the expression in transcripts per million (TPM). Expression of **a***GFOD1*, **b***KCNK17*, **c***KIF6*, **d***CBX2*, **e***LINC02078*, **f***ENPP7*, **g***TBC1D16*, **h***CARD14*

To shed light on the biological mechanisms regulated by the regulatory regions, the genic regulatory targets were sought to be identified. The CpG island near the *PHACTR1* migraine risk locus is overlapping the promoter region of various splice variants of the *GFOD1* gene and the non-coding *GFOD1-AS1* gene (Fig. 2a). We also found two *cis*-eQTLs within the CpG island that associated with *GFOD1* transcript levels (supplementary Table 4). Analysis of the gene expression in the GTEx database indicates that *GFOD1* is expressed across different kinds of tissue and is highly expressed in brain and vascular tissue (Fig. 3a).

PREs can be situated far away from their target genes, in introns, or in the 3′ untranslated regions (UTRs) [30]. Thus, the relations of the PREs to genes are based on *cis*-eQTLs in the GTEx database. Significant *cis*-eQTLs in the PRE near the *KCNK5* locus mapped to the *KCNK17* and *KIF6* gene (supplementary Table 4). The PRE is situated across two introns and one exon of the *KCNK17* gene (Fig. 2b). According to GTEx, *KCNK17* is primarily expressed in vascular tissue (Fig. 3b) while *KIF6* is mainly expressed in the brain (Fig. 3c).

The PRE near the *RNF213* locus harbored *cis*-eQTLs that associated with the transcript levels of five different genes. These include the nearby genes of *CBX2*, *LINC0207, ENPP7*, *TBC1D16*, and *CARD14* (Fig. 2c). We do not note a higher expression of these genes in brain or vascular tissue compared to other tissue types (Fig. 3d–h).

The PRE situated near the *ASTN2* locus is located in an intron of the *PAPPA* gene (Fig. 2d). No significant *cis*-eQTLs could be found within this PRE.

### No increased burden of rare variants in regulatory targets

We assessed whether the possible gene targets of the four regulatory regions had an increased burden of rare variants segregating with migraine. Using a familial association analysis, we did not find a significantly increased burden of rare variants segregating with migraine after Bonferroni correction (Table 3). The analysis was performed on the protein-coding genes only.

**Table 3:** Results from familial association analysis on rare variants segregating with migraine in the possible genic regulatory targets. The name of the migraine risk loci, the regulatory regions, and the corresponding possible genic regulatory target are presented. Additionally, the nominal *p* value and the Bonferroni-corrected *p* value of the familial association analysis are displayed

Locus	Type	Gene target	Nominal *p* value	Corrected *p*-value
*PHACTR1*	CpG island	*GFOD1*	0.088	0.62
*KCNK5*	PRE	*KCNK17*	7.6e-3	0.053
		*KIF6*	0.014	0.098
*RNF213*	PRE	*CBX2*	0.049	0.34
		*ENPP7*	0.056	0.39
		*TBC1D16*	0.037	0.26
		*CARD14*	0.098	0.69

## Discussion

Migraine is most often an episodic disorder and can be affected by regulatory factors, such as hormones. It is therefore very likely that gene dysregulation plays a causal role in migraine etiology. Here, we have assessed the hypothesis that an increased burden of rare regulatory variants in known migraine risk loci can be associated with a risk of migraine. We also addressed whether the association is independent of the common risk variants in the loci. We found four regulatory regions with an OR > 1, in which an increased burden of rare variants is independently associated with migraine. We report eight possible regulatory target genes. These genes are different than those in which the index SNPs of the known migraine risk loci resides in.

The CpG island near the *PHACTR1* locus is overlapping the promoter region of various splice variants of the *GFOD1* gene and the non-coding *GFOD1-AS1* gene. As about 70% of all promoters are located in a CpG island-rich area [31], it is highly likely that this CpG island is involved in the regulation of the *GFOD1* and the *GFOD1-AS1* gene. The regulation of the *GFOD1* gene is supported by the existence of two *cis*-eQTLs within the CpG island, that are associated with *GFOD1* transcript levels. Interestingly, the research group of Lasky-Su et al. [32] has reported a SNP in an intron of the *GFOD1* gene that associated with ADHD, which is a comorbid disorder of migraine [33, 34]. Thus, the findings of this study could help elucidate the association between the disorders. Analysis of the gene expression in the GTEx database indicates that *GFOD1* is expressed predominantly in brain and vascular tissue, which are tissue types that have been connected with migraine [35, 36]. Transcription of antisense non-coding RNAs has been shown to participate in both *cis* and *trans* gene regulation [37], and it is thus possible that *GFOD1-AS1* plays an important regulatory role on *GFOD1*.

Variations within CpG islands have been shown to influence DNA methylation patterns. Depending on the increase or elimination of CpG dinucleotides, the mutations may lead to hyper- or hypomethylation and, therefore, an altered transcriptional activity [38]. If CpG islands are hypermethylated, they may cause a stable silencing of the gene. In contrast, a hypomethylation can result in an overexpression [39]. Therefore, such changes of the CpG sites with the CpG island could affect the expression of *GFOD1*.

Significant *cis*-eQTLs in the PRE near the *KCNK5* locus drive a change in the gene expression of *KCNK17* and *KIF6*. The *KCNK17* gene encodes a membrane protein and belongs to the family of two-pore domain potassium (K2P) channels. Of the same K2P channel subfamily is the protein product of the *KCNK5* gene [40], in which the index SNP defining the migraine risk locus resides. From a different subfamily is the *KCNK18* gene where a frameshift mutation has been found to segregate perfectly in families with migraine with aura [41]. Additionally, previously identified migraine-associated genes include genes encoding potassium channels [41, 42]. Cortical spreading depression has been associated with migraine attacks [4], and because cortical spreading depression is characterized by ion influxes and neuronal depolarization, genetic alterations affecting potassium channels could play a role in this. The findings of our study could support that migraine may be a channelopathy and that potassium ion channels influence the disorder.

The PRE near the *RNF213* locus harbored *cis*-eQTLs that act upon five different genes. These include the nearby genes of *CBX2*, *LINC0207, ENPP7*, *TBC1D16*, and *CARD14*. The protein encoded by the *CARD14* gene mediates the activation of transcription factor NF-κB [43]. NF-κB plays a part in induction of nitric oxide production [44], which has been implied as having a key causative role in migraine [45] and can induce headache in healthy subjects [46]. Therefore, inhibition of NF-κB has been proposed as a possible therapeutic approach to treat migraine [47].

PREs are a class of silencers that have a suppressive effect on gene expression, when polycomb group (PcG) proteins are bound to them. Mutations within a PRE could result in reduced silencer activity and therefore lead to an increased gene expression. If mutations within the PRE results in increased expression of *CARD14*, it could therefore cause an increased activation of NF-κB and thus influence migraine.

The *CBX2* gene encodes a subunit of the PRC1-like complex, which is a PcG protein. Thus, this PcG protein participates in suppression of gene expression through binding to PREs. Thus, if the expression of the *CBX2* gene is altered, it could result in altered gene regulation.

Aside from finding a connection between a PcG protein and migraine, we have also found associations between an increased burden of rare variants in three PREs and migraine. It is interesting, that we find multiple connections between an increased migraine risk and PREs and proteins that bind to them. In humans, PREs are known to be influential on human embryonic development and epigenetic memory [48] but the function and regulatory mechanism of action of mammalian PREs is still largely unknown [49]. It is therefore speculative, what effect PREs have on migraine. As more knowledge on PREs is gained in the future, we may be able to understand how their altered regulatory mechanism may affect migraine. PcG proteins have been suggested to act synergistically in order to silence gene expression [50]. Consequently, any mutated PcG binding site within a PRE may cause an altered phenotype. Future research could include determining if the PREs we find associated with migraine harbor PcG bindings sites that have been altered by the rare mutations.

Lastly, we addressed whether only the regulatory regions had a higher burden of rare variants segregating with migraine or if the genes possibly targeted by the regulatory regions had a higher burden of rare variants. We found that none of the protein-coding possible genic regulatory targets had a significantly increased burden of rare variants segregating with migraine. This suggests that a higher burden of rare variants in the regulatory regions, and not their respective regulatory targets, is implicated in the pathophysiology of migraine. This supports that migraine is influenced by gene dysregulation and that regulatory dysfunction has a crucial impact on the disorder.

## Conclusion

We report that families with familial clustering of migraine have an increased burden of rare variants segregating with migraine in regulatory regions. The regulatory regions are located near known migraine risk loci but display effects independent of the common variants defining the loci. The possible regulatory targets suggest different genes than those originally tagged by the index SNPs of the migraine loci. The findings support that gene dysregulation plays a crucial, if not causal, role in migraine etiology.

## Electronic supplementary material

Supplementary Figure 1 Q-Q plot of the test statistics from the familial association analysis. The shaded area surrounding the reference line is the 95% confidence interval of expected *p*-values under the null hypothesis. (PNG 1723 kb)

High Resolution Image (TIFF 8791 kb)

ESM 1 (PDF 292 kb)

ESM 2 (PDF 283 kb)

ESM 3 (PDF 580 kb)

ESM 4 (PDF 28 kb)

## Data Availability

Summary data supporting the conclusions of this article are available on request.

## Abbreviations

CI: Confidence interval
F-SKAT: Family Sequence Kernel Association Test
GWAS: Genome-wide association study
K2P: Two-pore domain potassium
MAF: Minor allele frequency
OR: Odds ratio
PcG: Polycomb group
PRE: Polycomb group response elements
TFBS: Transcription factor binding sites

## References

[1] Stovner LJ, Nichols E, Steiner TJ, Abd-Allah F, Abdelalim A, Al-Raddadi RM (2018). Global, regional, and national burden of migraine and tension-type headache, 1990–2016: a systematic analysis for the global burden of disease study 2016. Lancet Neurol.

[2] Stovner LJ, Andrée C (2008). Impact of headache in Europe: a review for the Eurolight project on behalf of the Eurolight steering committee. J Headache Pain.

[3] GBD 2016 Disease and Injury Incidence and Prevalence Collaborators (2017). Global, regional, and national incidence, prevalence, and years lived with disability for 328 diseases and injuries for 195 countries, 1990–2016: a systematic analysis for the Global Burd. Lancet.

[4] (2018) Headache Classification Committee of the International Headache Society (IHS) The International Classification of Headache Disorders, 3rd edition. Cephalalgia 38(1):1–211. 10.1177/0333102417738202 10.1177/0333102417738202 29368949

[5] Hansen RD, Christensen AF, Olesen J (2017). Family studies to find rare high risk variants in migraine. J Headache Pain..

[6] Gormley P, Anttila V, Winsvold BS, Palta P, Esko T, Pers TH, Farh KH, Cuenca-Leon E, Muona M, Furlotte NA, Kurth T, Ingason A, McMahon G, Ligthart L, Terwindt GM, Kallela M, Freilinger TM, Ran C, Gordon SG, Stam AH, Steinberg S, Borck G, Koiranen M, Quaye L, Adams HH, Lehtimäki T, Sarin AP, Wedenoja J, Hinds DA, Buring JE, Schürks M, Ridker PM, Hrafnsdottir MG, Stefansson H, Ring SM, Hottenga JJ, Penninx BW, Färkkilä M, Artto V, Kaunisto M, Vepsäläinen S, Malik R, Heath AC, Madden PA, Martin NG, Montgomery GW, Kurki MI, Kals M, Mägi R, Pärn K, Hämäläinen E, Huang H, Byrnes AE, Franke L, Huang J, Stergiakouli E, Lee PH, Sandor C, Webber C, Cader Z, Muller-Myhsok B, Schreiber S, Meitinger T, Eriksson JG, Salomaa V, Heikkilä K, Loehrer E, Uitterlinden AG, Hofman A, van Duijn C, Cherkas L, Pedersen LM, Stubhaug A, Nielsen CS, Männikkö M, Mihailov E, Milani L, Göbel H, Esserlind AL, Christensen AF, Hansen TF, Werge T, Kaprio J, Aromaa AJ, Raitakari O, Ikram MA, Spector T, Järvelin MR, Metspalu A, Kubisch C, Strachan DP, Ferrari MD, Belin AC, Dichgans M, Wessman M, van den Maagdenberg A, Zwart JA, Boomsma DI, Smith GD, Stefansson K, Eriksson N, Daly MJ, Neale BM, Olesen J, Chasman DI, Nyholt DR, Palotie A, International Headache Genetics Consortium (2016). Meta-analysis of 375,000 individuals identifies 38 susceptibility loci for migraine. Nat Genet.

[7] Schork NJ, Murray SS, Frazer KA, Topol EJ (2009). Common vs. rare allele hypotheses for complex diseases. Curr Opin Genet Dev.

[8] Nicolae DL, Gamazon E, Zhang W, Duan S, Dolan ME, Cox NJ (2010). Trait-associated SNPs are more likely to be eQTLs: annotation to enhance discovery from GWAS. PLoS Genet.

[9] Maurano MT, Humbert R, Rynes E, Thurman RE, Haugen E, Wang H, Reynolds AP, Sandstrom R, Qu H, Brody J, Shafer A, Neri F, Lee K, Kutyavin T, Stehling-Sun S, Johnson AK, Canfield TK, Giste E, Diegel M, Bates D, Hansen RS, Neph S, Sabo PJ, Heimfeld S, Raubitschek A, Ziegler S, Cotsapas C, Sotoodehnia N, Glass I, Sunyaev SR, Kaul R, Stamatoyannopoulos JA (2012). Systematic localization of common disease-associated variation in regulatory DNA. Science..

[10] Aegidius K, Zwart J-A, Hagen K, Stovner L (2009). The effect of pregnancy and parity on headache prevalence: the head-HUNT study. Headache..

[11] Karli N, Baykan B, Ertas M, Zarifoglu M, Siva A, Saip S (2012). Impact of sex hormonal changes on tension-type headache and migraine: a cross-sectional population-based survey in 2,600 women. J Headache Pain..

[12] Kotani K, Shimomura T, Shimomura F, Ikawa S, Nanba E (2002). A polymorphism in the serotonin transporter gene regulatory region and frequency of migraine attacks. Headache..

[13] Formicola D, Aloia A, Sampaolo S, Farina O, Diodato D, Griffiths LR, Gianfrancesco F, di Iorio G, Esposito T (2010). Common variants in the regulative regions of GRIA1 and GRIA3 receptor genes are associated with migraine susceptibility. BMC Med Genet.

[14] Ravn J, Chalmer MA, Oehrstroem EL, Kogelman LJA, Hansen TF (2019) Characterization of familial and sporadic migraine. Headache. 10.1111/head.13640 10.1111/head.13640 PMC6899493 31544229

[15] Gervil M, Ulrich V, Olesen J, Russell MB (1998). Screening for migraine in the general population: validation of a simple questionnaire. Cephalalgia..

[16] Rasmussen BK, Jensen R, Olesen J (1991). Questionnaire versus clinical interview in the diagnosis of headache. Headache..

[17] Nissen L, Winther S, Isaksen C, Ejlersen JA, Brix L, Urbonaviciene G, Frost L, Madsen LH, Knudsen LL, Schmidt SE, Holm NR, Maeng M, Nyegaard M, Bøtker HE, Bøttcher M (2016). Danish study of non-invasive testing in coronary artery disease (Dan-NICAD): study protocol for a randomised controlled trial. Trials..

[18] Jonsson H, Sulem P, Kehr B, Kristmundsdottir S, Zink F, Hjartarson E (2017). Whole genome characterization of sequence diversity of 15,220 Icelanders. Sci data.

[19] Karolchik D, Hinrichs AS, Furey TS, Roskin KM, Sugnet CW, Haussler D, Kent WJ (2004). The UCSC table browser data retrieval tool. Nucleic Acids Res.

[20] Ernst J, Kellis M (2010). Discovery and characterization of chromatin states for systematic annotation of the human genome. Nat Biotechnol.

[21] Gerstein MB, Kundaje A, Hariharan M, Landt SG, Yan KK, Cheng C, Mu XJ, Khurana E, Rozowsky J, Alexander R, Min R, Alves P, Abyzov A, Addleman N, Bhardwaj N, Boyle AP, Cayting P, Charos A, Chen DZ, Cheng Y, Clarke D, Eastman C, Euskirchen G, Frietze S, Fu Y, Gertz J, Grubert F, Harmanci A, Jain P, Kasowski M, Lacroute P, Leng JJ, Lian J, Monahan H, O'Geen H, Ouyang Z, Partridge EC, Patacsil D, Pauli F, Raha D, Ramirez L, Reddy TE, Reed B, Shi M, Slifer T, Wang J, Wu L, Yang X, Yip KY, Zilberman-Schapira G, Batzoglou S, Sidow A, Farnham PJ, Myers RM, Weissman SM, Snyder M (2012). Architecture of the human regulatory network derived from ENCODE data. Nature..

[22] Fishilevich S, Nudel R, Rappaport N, Hadar R, Plaschkes I, Iny Stein T et al (2017) GeneHancer: genome-wide integration of enhancers and target genes in GeneCards. Database (Oxford). 10.1093/database/bax028 10.1093/database/bax028 PMC5467550 28605766

[23] Gardiner-Garden M, Frommer M (1987). CpG islands in vertebrate genomes. J Mol Biol.

[24] Dreos R, Ambrosini G, Cavin Perier R, Bucher P (2013). EPD and EPDnew, high-quality promoter resources in the next-generation sequencing era. Nucleic Acids Res.

[25] Yan Q, Tiwari HK, Yi N, Gao G, Zhang K, Lin WY, Lou XY, Cui X, Liu N (2015). A sequence kernel association test for dichotomous traits in family samples under a generalized linear mixed model. Hum Hered.

[26] Clarke GM, Anderson CA, Pettersson FH, Cardon LR, Morris AP, Zondervan KT (2011). Basic statistical analysis in genetic case-control studies. Nat Protoc.

[27] Sherry ST, Ward MH, Kholodov M, Baker J, Phan L, Smigielski EM, Sirotkin K (2001). dbSNP: the NCBI database of genetic variation. Nucleic Acids Res.

[28] Mathieson I, McVean G (2012). Differential confounding of rare and common variants in spatially structured populations. Nat Genet.

[29] Pirie A, Wood A, Lush M, Tyrer J, Pharoah PD (2015). The effect of rare variants on inflation of the test statistics in case-control analyses. BMC Bioinformatics.

[30] Maston GA, Evans SK, Green MR (2006). Transcriptional regulatory elements in the human genome. Annu Rev Genomics Hum Genet.

[31] Saxonov S, Berg P, Brutlag DL (2006). A genome-wide analysis of CpG dinucleotides in the human genome distinguishes two distinct classes of promoters. Proc Natl Acad Sci U S A.

[32] Lasky-Su J, Neale BM, Franke B, Anney RJ, Zhou K, Maller JB (2008). Genome-wide association scan of quantitative traits for attention deficit hyperactivity disorder identifies novel associations and confirms candidate gene associations. Am J Med Genet B Neuropsychiatr Genet.

[33] Hansen TF, Hoeffding LK, Kogelman L, Haspang TM, Ullum H, Sorensen E (2018). Comorbidity of migraine with ADHD in adults. BMC Neurol.

[34] Fasmer OB, Halmoy A, Oedegaard KJ, Haavik J (2011). Adult attention deficit hyperactivity disorder is associated with migraine headaches. Eur Arch Psychiatry Clin Neurosci.

[35] Tfelt-Hansen PC, Koehler PJ (2011). One hundred years of migraine research: major clinical and scientific observations from 1910 to 2010. Headache..

[36] Pietrobon D, Striessnig J (2003). Neurobiology of migraine. Nat Rev Neurosci.

[37] Villegas VE, Zaphiropoulos PG (2015). Neighboring gene regulation by antisense long non-coding RNAs. Int J Mol Sci.

[38] Samy MD, Yavorski JM, Mauro JA, Blanck G (2016). Impact of SNPs on CpG Islands in the MYC and HRAS oncogenes and in a wide variety of tumor suppressor genes: a multi-cancer approach. Cell Cycle.

[39] Bird A (2002). DNA methylation patterns and epigenetic memory. Genes Dev.

[40] Enyedi P, Czirjak G (2010). Molecular background of leak K+ currents: two-pore domain potassium channels. Physiol Rev.

[41] Lafreniere RG, Cader MZ, Poulin JF, Andres-Enguix I, Simoneau M, Gupta N (2010). A dominant-negative mutation in the TRESK potassium channel is linked to familial migraine with aura. Nat Med.

[42] Cox HC, Lea RA, Bellis C, Carless M, Dyer T, Blangero J, Griffiths LR (2011). Variants in the human potassium channel gene (KCNN3) are associated with migraine in a high risk genetic isolate. J Headache Pain.

[43] Bertin J, Wang L, Guo Y, Jacobson MD, Poyet JL, Srinivasula SM, Merriam S, DiStefano P, Alnemri ES (2001) CARD11 and CARD14 are novel caspase recruitment domain (CARD)/membrane-associated guanylate kinase (MAGUK) family members that interact with BCL10 and activate NF-kappa B. J Biol Chem 276(15):11877–11882. 10.1074/jbc.M010512200 10.1074/jbc.M010512200 11278692

[44] Xie QW, Kashiwabara Y, Nathan C (1994). Role of transcription factor NF-kappa B/Rel in induction of nitric oxide synthase. J Biol Chem.

[45] Olesen J, Iversen HK, Thomsen LL (1993). Nitric oxide supersensitivity: a possible molecular mechanism of migraine pain. Neuroreport..

[46] Iversen HK, Olesen J (1996). Headache induced by a nitric oxide donor (nitroglycerin) responds to sumatriptan. A human model for development of migraine drugs. Cephalalgia..

[47] Reuter U, Chiarugi A, Bolay H, Moskowitz MA (2002). Nuclear factor-kappaB as a molecular target for migraine therapy. Ann Neurol.

[48] Schmitt S, Prestel M, Paro R (2005). Intergenic transcription through a polycomb group response element counteracts silencing. Genes Dev.

[49] Bauer M, Trupke J, Ringrose L (2016). The quest for mammalian Polycomb response elements: are we there yet?. Chromosoma..

[50] Breen TR, Duncan IM (1986). Maternal expression of genes that regulate the bithorax complex of Drosophila melanogaster. Dev Biol.

