# Supplementary figures and images for: Familial analysis reveals rare risk variants for migraine in regulatory regions
Source: Neurogenetics. 2020 Feb 19;21(3):149–57. doi: 10.1007/s10048-020-00606-5 (PMC7283211; doi:10.1007/s10048-020-00606-5)

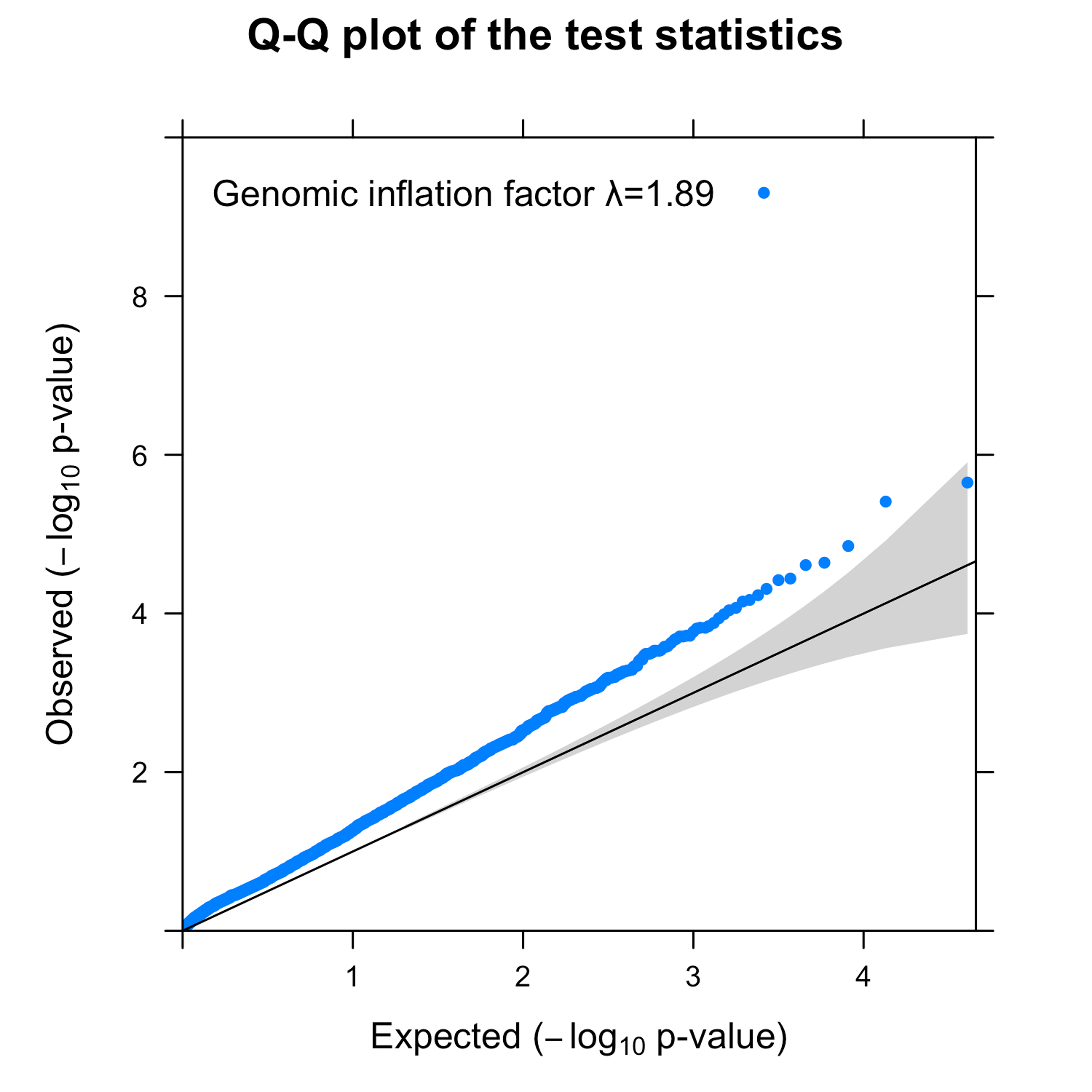

Supplement: Supplementary file 1 — Q-Q plot of the test statistics from the familial association analysis. The shaded area surrounding the reference line is the 95% confidence interval of expected p-values under the null hypothesis. (PNG 1723 kb) [file 10048_2020_606_Fig4_ESM.png]

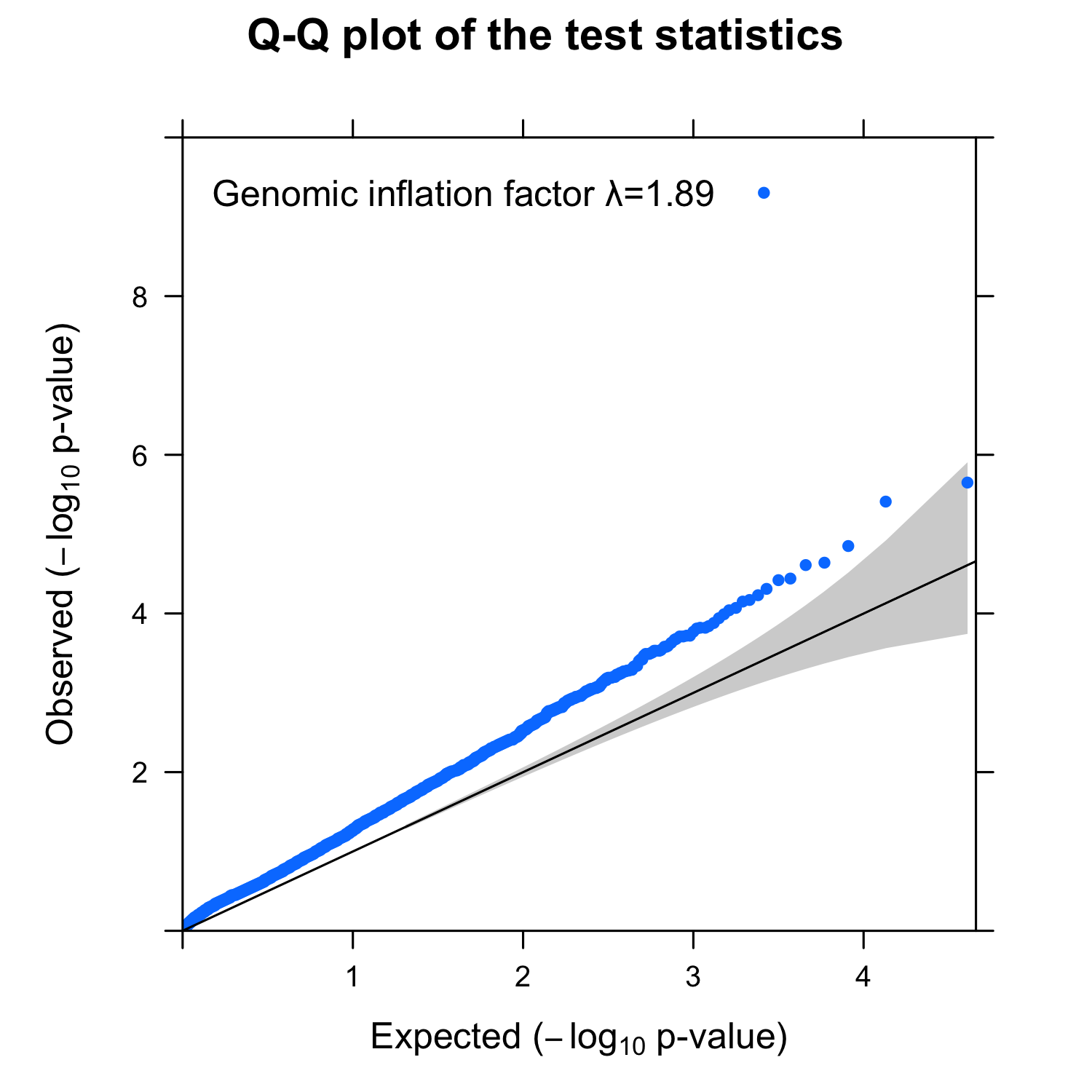

Supplement: Supplementary file 2 — High Resolution Image (TIFF 8791 kb) [file 10048_2020_606_MOESM1_ESM.tiff]
