# Supplementary material for: Familial analysis reveals rare risk variants for migraine in regulatory regions
Source: Neurogenetics. 2020 Feb 19;21(3):149–57. doi: 10.1007/s10048-020-00606-5 (PMC7283211; doi:10.1007/s10048-020-00606-5)
Supplement: Supplementary file 3 — (PDF 292 kb) [file 10048_2020_606_MOESM2_ESM.pdf]

Article title: Familial analysis reveals rare risk variants for migraine in regulatory regions

Journal name: Neurogenetics

Tanya Techlo[1], Andreas Høiberg Rasmussen[1], Peter L. Møller[2], Morten Bøttcher[3], Simon Winther[3,4], Olafur B. Davidsson[1], Isa A. Olofsson[1], Mona Ameri Chalmer[1], Lisette J. A. Kogelman[1], Mette Nyegaard[2], Jes Olesen[1], Thomas Folkmann Hansen*[1,5,6]

[1]Danish Headache Center, Department of Neurology, Rigshospitalet Glostrup, Glostrup, Denmark.

[2]Department of Biomedicine, Hoegh-Guldbergsgade 10, Aarhus University, Aarhus, Denmark

[3]Department of Cardiology, Hospital Unit West Jutland, Herning, Denmark

[4]Department of Cardiology, Aarhus University Hospital, Skejby, Aarhus, Denmark

[5]Institute for Biological Psychiatry, Mental Health Center Sct. Hans, Denmark

[6]Novo Nordic Foundation Centre for Protein Research, Copenhagen University, Copenhagen, Denmark

***Corresponding author:**

Thomas Folkmann Hansen, Danish Headache Center, Department of Neurology, Rigshospitalet Glostrup, Nordstjernevej 40 DK-2600 Glostrup, Denmark.

Phone: +45 38633051, Email: thomas.hansen@regionh.dk

Supplementary table 1 The autosomal migraine risk loci with corresponding genomic areas analyzed in this study. The table displays the loci names, SNP identifiers, and MAF of the index SNPs for autosomal migraine risk loci, the genomic positions of the index SNPs, and the analyzed genomic areas.

Locus	SNP identifier	MAF	Position of index SNP (chromosome:position)	Analyzed genomic area (chromosome:start:end)
PRDM16	rs10218452	0.22	chr1:3159033	chr1:2159033:4159033
1p31.1	rs1572668	0.48	chr1:73434059	chr1:72434059:74434059
Near *TSPAN2-NGF*	rs2078371	0.12	chr1:115134562	chr1:114134562:116134562
Near *ADAMTSL4-ECM1*	rs6693567	0.27	chr1:150538184	chr1:149538184:151538184
MEF2D	rs1925950	0.35	chr1:156480948	chr1:155480948:157480948
CARF	rs138556413	0.03	chr2:202968144	chr2:201968144:203968144
TRPM8-HJURP	rs10166942	0.20	chr2:233916448	chr2:232916448:234916448
Near *TGFBR2*	rs6791480	0.31	chr3:30439067	chr3:29439067:31439067
Near *GPR149*	rs13078967	0.03	chr3:154572157	chr3:153572157:155572157
Near *REST-SPINK2*	rs7684253	0.45	chr4:56861145	chr4:55861145:57861145
PHACTR1	rs9349379	0.41	chr6:12903725	chr6:11903725:13903725
Near *NOTCH4*	rs140002913	0.06	chr6:32238272	chr6:31238272:33238272
KCNK5	rs10456100	0.28	chr6:39215694	chr6:38215694:40215694
FHL5-UFL1	rs67338227	0.23	chr6:96594271	chr6:95594271:97594271
Near *GJA1*	rs28455731	0.16	chr6:121524892	chr6:120524892:122524892
HEY2-NCOA7	rs1268083	0.48	chr6:125727894	chr6:124727894:126727894
C7orf10	rs186166891	0.11	chr7:40367277	chr7:39367277:41367277
Near *DOCK4-IMMP2L*	rs10155855	0.05	chr7:111688341	chr7:110688341:112688341
ASTN2	rs6478241	0.36	chr9:116490350	chr9:115490350:117490350
NRP1	rs2506142	0.17	chr10:33179196	chr10:32179196:34179196
PLCE1	rs10786156	0.45	chr10:94254865	chr10:93254865:95254865

HPSE2	rs12260159	0.07	chr10:98942980	chr10:97942980:99942980
ARMS2-HTRA1	rs2223089	0.08	chr10:122450644	chr10:121450644:123450644
MRVI1	rs4910165	0.33	chr11:10652497	chr11:9652497:11652497
MPPED2	rs11031122	0.24	chr11:30525891	chr11:29525891:31525891
YAP1	rs10895275	0.33	chr11:102212877	chr11:101212877:103212877
IGSF9B	rs561561	0.12	chr11:133959811	chr11:132959811:134959811
Near *FGF6*	rs1024905	0.47	chr12:4408974	chr12:3408974:5408974
LRP1-STAT6-SDR9C7	rs11172113	0.42	chr12:57133500	chr12:56133500:58133500
Near *ITPK1*	rs11624776	0.31	chr14:93129246	chr14:92129246:94129246
CFDP1	rs77505915	0.45	chr16:75408245	chr16:74408245:76408245
Near *ZCCHC14*	rs4081947	0.34	chr16:87546264	chr16:86546264:88546264
Near *WSCD1-NLRP1*	rs75213074	0.03	chr17:5709320	chr17:4709320:6709320
RNF213	rs17857135	0.17	chr17:80288362	chr17:79288362:81288362
Near *JAG1*	rs111404218	0.34	chr20:10703511	chr20:9703511:11703511
SLC24A3	rs4814864	0.26	chr20:19489173	chr20:18489173:20489173
Near *CCM2L-HCK*	rs144017103	0.02	chr20:32041179	chr20:31041179:33041179
