# Supplementary material for: Familial analysis reveals rare risk variants for migraine in regulatory regions
Source: Neurogenetics. 2020 Feb 19;21(3):149–57. doi: 10.1007/s10048-020-00606-5 (PMC7283211; doi:10.1007/s10048-020-00606-5)
Supplement: Supplementary file 4 — (PDF 283 kb) [file 10048_2020_606_MOESM3_ESM.pdf]

Article title: Familial analysis reveals rare risk variants for migraine in regulatory regions

Journal name: Neurogenetics

Tanya Techlo[1], Andreas Høiberg Rasmussen[1], Peter L. Møller[2], Morten Bøttcher[3], Simon Winther[3,4], Olafur B. Davidsson[1], Isa A. Olofsson[1], Mona Ameri Chalmer[1], Lisette J. A. Kogelman[1], Mette Nyegaard[2], Jes Olesen[1], Thomas Folkmann Hansen*[1,5,6]

[1]Danish Headache Center, Department of Neurology, Rigshospitalet Glostrup, Glostrup, Denmark.

[2]Department of Biomedicine, Hoegh-Guldbergsgade 10, Aarhus University, Aarhus, Denmark

[3]Department of Cardiology, Hospital Unit West Jutland, Herning, Denmark

[4]Department of Cardiology, Aarhus University Hospital, Skejby, Aarhus, Denmark

[5]Institute for Biological Psychiatry, Mental Health Center Sct. Hans, Denmark

[6]Novo Nordic Foundation Centre for Protein Research, Copenhagen University, Copenhagen, Denmark

***Corresponding author:**

Thomas Folkmann Hansen, Danish Headache Center, Department of Neurology, Rigshospitalet Glostrup, Nordstjernevej 40 DK-2600 Glostrup, Denmark.

Phone: +45 38633051, Email: thomas.hansen@regionh.dk

Supplementary table 2. Summary of the results obtained from the familial association analysis. The table gives the name of the migraine risk loci, the number of regulatory regions per locus with a significant increased burden, the genomic positions of the regulatory regions, the type of regulatory regions, and the Bonferroni-corrected *p*-value.

Locus	Number of regulatory regions	Position (chromosome:start:end)	Type	*p*-value
PRDM16	1	chr1:3585916:3586226	TFBS	0.043
MEF2D	3	chr1:156243980:156248680	Enhancer	0.011
		chr1:156245314:156248566	TFBS	0.048
		chr1:156490646:156490706	Promoter	0.046
CARF	5	chr2:202018973:202019707	Enhancer	0.042
		chr2:202030311:202040075	Enhancer	0.011
		chr2:202632257:202638540	Enhancer	0.029
		chr2:202032663:202036149	CpG island	$4.2 \cdot 10^{-3}$
		chr2:202060232:202060832	Insulator	$1.5 \cdot 10^{-3}$
Near *GPR149*	1	chr3:153957426:153957670	TFBS	0.011
Near *REST-SPINK2*	3	chr4:56212277:56215077	PRE	$5.6 \cdot 10^{-5}$
		chr4:56505416:56505856	CpG island	0.026
		chr4:56904877:56906077	PRE	$3.3 \cdot 10^{-3}$
PHACTR1	1	chr6:13486092:13488560	CpG island	0.044
Near *NOTCH4*	1	chr6:31652354:31652414	Promoter	0.044
KCNK5	1	chr6:39310446:39312846	PRE	0.011
ASTN2	2	chr9:116284900:116285300	PRE	0.011
		chr9:117488447:117488857	TFBS	$1.3 \cdot 10^{-3}$
PLCE1	1	chr10:94005223:94008418	Enhancer	0.017
HPSE2	1	chr10:99840130:99840469	CpG island	0.017
MRVI1	1	chr11:11235277:11237077	PRE	0.026

MPPED2	1	chr11:30582477:30583677	PRE	0.036
Near *FGF6*	1	chr12:3489902:3490712	TFBS	0.014
LRP1-STAT6-SDR9C7	2	chr12:56718130:56726985	Enhancer	$6.0 \cdot 10^{-3}$
		chr12:56721185:56721850	TFBS	0.046
CFDP1	2	chr16:74775601:74776201	PRE	0.018
		chr16:75799001:75799201	Insulator	$3.2 \cdot 10^{-4}$
Near *ZCCHC14*	2	chr16:88502859:88503083	CpG island	0.046
		chr16:88538891:88539691	PRE	0.038
RNF213	5	chr17:79742606:79746206	PRE	$5.7 \cdot 10^{-3}$
		chr17:79745858:79748878	Enhancer	0.025
		chr17:79774606:79774806	Insulator	$1.4 \cdot 10^{-3}$
		chr17:79923138:79934231	Enhancer	0.048
		chr17:79942885:79943117	CpG island	0.028
Near *JAG1*	1	chr20:10224244:10224304	Promoter	0.022
SLC24A3	2	chr20:19462556:19462956	Insulator	0.047
		chr20:19918942:19919342	TFBS	0.016
Near *CCM2L-HCK*	2	chr20:32316663:32317179	TFBS	0.039
		chr20:32496934:32497344	TFBS	0.013
