# Supplementary material for: Familial analysis reveals rare risk variants for migraine in regulatory regions
Source: Neurogenetics. 2020 Feb 19;21(3):149–57. doi: 10.1007/s10048-020-00606-5 (PMC7283211; doi:10.1007/s10048-020-00606-5)
Supplement: Supplementary file 5 — (PDF 580 kb) [file 10048_2020_606_MOESM4_ESM.pdf]

Supplementary table 3. Information on the rare risk variants identified for the four regulatory regions that were verified in the replication cohort. The table is divided into sections by regulatory region. Each section contains information on the rare variants within the regulatory region, including the genomic position, reference and alternative allele, available SNP identifiers, and the MAF in the families. Additionally is displayed the variant consequence, transcript biotype, and affected gene as predicted by Ensembl Variant Effect Predictor.

**CpG island on chromosome 6 spanning position 13486092-13488560**									
**Variant position**	**Reference allele**	**Alternative allele**	**SNP identifier**	**MAF in cases**	**MAF in controls**	**Cohort MAF**	**Consequence**	**Biotype**	**Gene**
13486207	T	C		0	1.7e-03	4.8e-04	Intron variant	Protein coding	GFOD1
							Regulatory region variant	Promoter	
13486211	A	C		6.7e-04	0	4.8e-04	Intron variant	Protein coding	GFOD1
							Regulatory region variant	Promoter	
13486225	A	C	rs1156912071	6.7e-04	0	4.8e-04	Intron variant	Protein coding	GFOD1
							Regulatory region variant	Promoter	
13486232	T	C	rs1009132982	2.7e-03	3.4e-03	2.9e-03	Intron variant	Protein coding	GFOD1
							Regulatory region variant	Promoter	
13486235	T	C	rs1019642032	3.4e-03	5.1e-03	3.9e-03	Intron variant	Protein coding	GFOD1

							Regulatory region variant	Promoter	
13486238	A	C	rs965006702	6.7e-04	0	4.8e-04	Intron variant	Protein coding	GFOD1
							Regulatory region variant	Promoter	
13486243	C	A	rs992961184	0	1.7e-03	4.8e-04	Intron variant	Protein coding	GFOD1
							Regulatory region variant	Promoter	
13486245	CAT	C	rs1491121689	2.0e-02	2.2e-02	2.1e-02	Intron variant	Protein coding	GFOD1
							Regulatory region variant	Promoter	
13486246	AT	A	rs1163853849	0	1.7e-03	4.8e-04	Intron variant	Protein coding	GFOD1
							Regulatory region variant	Promoter	
13486246	AT	ACT		6.7e-04	0	4.8e-04	Intron variant	Protein coding	GFOD1
							Regulatory region variant	Promoter	
13486246	A	C	rs999187989	9.4e-03	2.2e-02	1.3e-02	Intron variant	Protein coding	GFOD1
							Regulatory region variant	Promoter	
13486246	A	T	rs999187989	0	1.7e-03	4.8e-04	Intron variant	Protein coding	GFOD1
							Regulatory region variant	Promoter	
13486247	T	TC	rs1187872279	5.4e-03	8.5e-03	6.3e-03	Intron variant	Protein coding	GFOD1

							Regulatory region variant	Promoter	
13486247	T	TCCC	rs1187872279	3.4e-03	0	2.4e-03	Intron variant	Protein coding	GFOD1
							Regulatory region variant	Promoter	
13486247	T	TCCCCCCCCCC C		0	1.7e-03	4.8e-04	Intron variant	Protein coding	GFOD1
							Regulatory region variant	Promoter	
13486247	T	TCCCCCCCCCC CC		1.3e-03	0	9.6e-04	Intron variant	Protein coding	GFOD1
							Regulatory region variant	Promoter	
13486247	T	TCCCCCCCCCC CCC		3.4e-03	5.1e-03	3.9e-03	Intron variant	Protein coding	GFOD1
							Regulatory region variant	Promoter	
13486247	T	TCCCCCCCCCC CCCC		6.7e-04	0	4.8e-04	Intron variant	Protein coding	GFOD1
							Regulatory region variant	Promoter	
13486247	T	TCCCCCCCCCC CCCCC		6.7e-04	0	4.8e-04	Intron variant	Protein coding	GFOD1
							Regulatory region variant	Promoter	
13486247	T	TCCCCCCCCCC CCCCCC		1.3e-03	1.7e-03	1.4e-03	Intron variant	Protein coding	GFOD1
							Regulatory region variant	Promoter	
13486247	T	TCCCCCCCCCC CCCCCCC		0	1.7e-03	4.8e-04	Intron variant	Protein coding	GFOD1

							Regulatory region variant	Promoter	
13486247	T	TCCCCCCCCCC CCCCCCCC		6.7e-04	1.7e-03	9.6e-04	Intron variant	Protein coding	GFOD1
							Regulatory region variant	Promoter	
13486247	T	TCCCCCCCCCC CCCCCCCCC		6.7e-04	0	4.8e-04	Intron variant	Protein coding	GFOD1
							Regulatory region variant	Promoter	
13486247	T	TCCCCCCCCCC CCCCCCCCCC		0	1.7e-03	4.8e-04	Intron variant	Protein coding	GFOD1
							Regulatory region variant	Promoter	
13486247	T	TCCCCCCCCCC CCCCCCCCCCC CCCCCCCCCC		0	1.7e-03	4.8e-04	Intron variant	Protein coding	GFOD1
							Regulatory region variant	Promoter	
13486247	T	TCCCCCCCCCC CCCCCCCCCCC CCCCCCCCGC		0	1.7e-03	4.8e-04	Intron variant	Protein coding	GFOD1
							Regulatory region variant	Promoter	
13486247	TC	CC	rs1030626971	1.3e-02	8.5e-03	1.2e-02	Intron variant	Protein coding	GFOD1
							Regulatory region variant	Promoter	
13486247	TC	T	rs373896526	6.7e-04	0	4.8e-04	Intron variant	Protein coding	GFOD1
							Regulatory region variant	Promoter	

13486247	TC	TCC	rs1187872279	2.0e-03	0	1.4e-03	Intron variant	Protein coding	GFOD1
							Regulatory region variant	Promoter	
13486247	TC	TCCCCC		6.7e-04	0	4.8e-04	Intron variant	Protein coding	GFOD1
							Regulatory region variant	Promoter	
13486247	TC	TCCCCCCCCCCCCCCCC		1.3e-03	0	9.6e-04	Intron variant	Protein coding	GFOD1
							Regulatory region variant	Promoter	
13486247	TC	TCCCCCCCCCCCCC		6.7e-04	0	4.8e-04	Intron variant	Protein coding	GFOD1
							Regulatory region variant	Promoter	
13486247	TC	TCCCCCCCCCCCCCC		2.7e-03	0	1.9e-03	Intron variant	Protein coding	GFOD1
							Regulatory region variant	Promoter	
13486247	TC	TCCCCCCCCCCCCCCC		6.7e-04	0	4.8e-04	Intron variant	Protein coding	GFOD1
							Regulatory region variant	Promoter	
13486247	TCC	T	rs373896526	2.0e-03	0	1.4e-03	Intron variant	Protein coding	GFOD1
							Regulatory region variant	Promoter	
13486250	C	CCCCCCCCCCCCCCCCCCCCCCCCCG		6.7e-04	0	4.8e-04	Intron variant	Protein coding	GFOD1
							Regulatory region variant	Promoter	

13486255	C	T	rs1396460560	0	1.7e-03	4.8e-04	Intron variant	Protein coding	GFOD1
							Regulatory region variant	Promoter	
13486257	C	A	rs28392827	3.4e-03	6.8e-03	4.3e-03	Intron variant	Protein coding	GFOD1
							Regulatory region variant	Promoter	
13486258	C	A	rs57203835	1.1e-02	1.9e-02	1.3e-02	Intron variant	Protein coding	GFOD1
							Regulatory region variant	Promoter	
13486258	CA	C	rs375882366	2.7e-03	8.5e-03	4.3e-03	Intron variant	Protein coding	GFOD1
							Regulatory region variant	Promoter	
13486261	A	C	rs1252665552	4.7e-03	6.8e-03	5.3e-03	Intron variant	Protein coding	GFOD1
							Regulatory region variant	Promoter	
13486263	A	C	rs1419858552	6.7e-04	0	4.8e-04	Intron variant	Protein coding	GFOD1
							Regulatory region variant	Promoter	
13486297	G	C	rs2494772	2.7e-03	0	1.9e-03	Intron variant	Protein coding	GFOD1
							Regulatory region variant	Promoter	
							Noncoding transcript exon variant	Antisense	GFOD1-AS1
13486407	G	C	rs114976037	1.0e-02	1.9e-02	1.3e-02	Intron variant	Protein coding	GFOD1

							Regulatory region variant	Promoter	
							Regulatory region variant	CTCF binding site	
							Noncoding transcript exon variant	Antisense	GFOD1-AS1
13486494	C	A	rs1229633433	1.3e-03	0	9.6e-04	Intron variant	Protein coding	GFOD1
							Regulatory region variant	Promoter	
							Regulatory region variant	CTCF binding site	
							Noncoding transcript exon variant	Antisense	GFOD1-AS1
13486567	AG	A	rs1039121685	2.0e-03	0	1.4e-03	Intron variant	Protein coding	GFOD1
							Regulatory region variant	Promoter	
							Regulatory region variant	CTCF binding site	
							Noncoding transcript exon variant	Antisense	GFOD1-AS1
13486595	A	G	rs1241129285	6.7e-04	0	4.8e-04	Intron variant	Protein coding	GFOD1
							Regulatory region variant	Promoter	
							Regulatory region variant	CTCF binding site	

							Noncoding transcript exon variant	Antisense	GFOD1-AS1
13486605	T	G	rs533368418	1.3e-03	1.7e-03	1.4e-03	Intron variant	Protein coding	GFOD1
							Regulatory region variant	Promoter	
							Regulatory region variant	CTCF binding site	
							Noncoding transcript exon variant	Antisense	GFOD1-AS1
13486942	C	T	rs62385806	1.0e-02	1.9e-02	1.3e-02	5’ UTR variant	Protein coding	GFOD1
							Regulatory region variant	Promoter	
							Regulatory region variant	CTCF binding site	
13487079	G	A		1.3e-03	3.4e-03	1.9e-03	5’ UTR variant	Protein coding	GFOD1
							Regulatory region variant	Promoter	
							Regulatory region variant	CTCF binding site	
13487170	G	A	rs549568711	4.0e-03	3.4e-03	3.9e-03	5’ UTR variant	Protein coding	GFOD1
							Regulatory region variant	Promoter	
							Regulatory region variant	CTCF binding site	
							TFBS variant		
13487262	G	C		6.7e-04	1.7e-03	9.6e-04	5’ UTR variant	Protein coding	GFOD1

							Regulatory region variant	Promoter	
							Regulatory region variant	CTCF binding site	
13487320	G	T	rs982391	1.0e-02	1.9e-02	1.3e-02	5' UTR variant	Protein coding	GFOD1
							Regulatory region variant	Promoter	
							Regulatory region variant	CTCF binding site	
13487362	A	G	rs114234739	4.7e-03	3.4e-03	4.3e-03	5' UTR variant	Protein coding	GFOD1
							Regulatory region variant	Promoter	
							Regulatory region variant	CTCF binding site	
13487399	T	C	rs2494773	2.7e-03	0	1.9e-03	5' UTR variant	Protein coding	GFOD1
							Regulatory region variant	Promoter	
							Regulatory region variant	CTCF binding site	
13487778	C	T	rs550492110	1.1e-02	0	7.7e-03	Regulatory region variant	Promoter	
							Regulatory region variant	CTCF binding site	
							Intergenic variant		
13487934	T	C	rs552818164	1.3e-03	0	9.6e-04	Regulatory region variant	Promoter	

							Regulatory region variant	CTCF binding site	
							Intergenic variant		
13488369	A	C	rs1040627839	2.0e-03	3.4e-03	2.4e-03	Regulatory region variant	Promoter	
							Regulatory region variant	CTCF binding site	
							Intergenic variant		
13488372	T	C	rs903407134	6.7e-04	3.4e-03	1.4e-03	Regulatory region variant	Promoter	
							Regulatory region variant	CTCF binding site	
							Intergenic variant		
13488378	G	C	rs999070925	1.3e-03	1.0e-02	3.9e-03	Regulatory region variant	Promoter	
							Regulatory region variant	CTCF binding site	
							Intergenic variant		
13488383	T	C	rs1396741578	2.0e-03	1.0e-02	4.3e-03	Regulatory region variant	Promoter	
							Regulatory region variant	CTCF binding site	
							Intergenic variant		
13488390	A	C	rs888205423	1.3e-03	0	9.6e-04	Regulatory region variant	Promoter	

							Regulatory region variant	CTCF binding site	
							Intergenic variant		
13488433	G	A	rs933681258	1.3e-03	0	9.6e-04	Regulatory region variant	Promoter	
							Regulatory region variant	CTCF binding site	
							Intergenic variant		
13488444	T	G	rs1261685243	6.7e-04	0	4.8e-04	Regulatory region variant	Promoter	
							Regulatory region variant	CTCF binding site	
							Intergenic variant		
13488449	G	C	rs1050765996	6.7e-04	0	4.8e-04	Regulatory region variant	Promoter	
							Regulatory region variant	CTCF binding site	
							Intergenic variant		
13488481	G	C		6.7e-04	0	4.8e-04	Regulatory region variant	Promoter	
							Regulatory region variant	CTCF binding site	
							Intergenic variant		
13488490	C	A	rs542425479	6.0e-03	3.4e-03	5.3e-03	Regulatory region variant	Promoter	

							Regulatory region variant	CTCF binding site	
							Intergenic variant		
**PRE on chromosome 6 spanning position 39310446-39312846**									
**Variant position**	**Reference allele**	**Alternative allele**	**SNP identifier**	**MAF in cases**	**MAF in controls**	**Cohort MAF**	**Consequence**	**Biotype**	**Gene**
39310523	C	A	rs12196391	5.4e-03	1.0e-02	6.7e-03	Intron variant	Protein coding	KCNK17
							Noncoding transcript exon variant	Retained intron	KCNK17
							Regulatory region variant	CTCF binding site	
39310607	G	A	rs571544467	6.7e-04	0	4.8e-04	Intron variant	Protein coding	KCNK17
							Noncoding transcript exon variant	Retained intron	KCNK17
							Regulatory region variant	CTCF binding site	
39310675	T	C		6.7e-04	0	4.8e-04	Intron variant	Protein coding	KCNK17
							Noncoding transcript exon variant	Retained intron	KCNK17
							Regulatory region variant	CTCF binding site	
39310729	T	C	rs116276161	7.4e-03	3.4e-03	6.3e-03	Intron variant	Protein coding	KCNK17
							Noncoding transcript exon variant	Retained intron	KCNK17

							Regulatory region variant	CTCF binding site	
39310918	AAAG	A	rs540653476, COSM5357017, COSM5357018	1.1e-02	1.5e-02	1.2e-02	Noncoding transcript exon variant	Retained intron	KCNK17
							Inframe deletion	Protein coding	KCNK17
							Regulatory region variant	CTCF binding site	
39310980	C	T	rs143146161, COSM1329694, COSM1329695	1.3e-03	1.7e-03	1.4e-03	Noncoding transcript exon variant	Retained intron	KCNK17
							Missense variant	Protein coding	KCNK17
							Regulatory region variant	CTCF binding site	
39311018	G	C	rs72855550	6.7e-04	0	4.8e-04	Intron variant	Protein coding	KCNK17
							Noncoding transcript exon variant	Retained intron	KCNK17
							Regulatory region variant	CTCF binding site	
39311050	T	C	rs9471060	6.7e-04	1.7e-03	9.6e-04	Intron variant	Protein coding	KCNK17
							Noncoding transcript exon variant	Retained intron	KCNK17
							Regulatory region variant	CTCF binding site	
39311072	G	GCA	rs1373643967	1.1e-02	1.0e-02	1.1e-02	Intron variant	Protein coding	KCNK17
							Noncoding transcript exon variant	Retained intron	KCNK17

							Regulatory region variant	CTCF binding site	
39311072	G	GCACA	rs1373643967	1.9e-02	1.7e-02	1.8e-02	Intron variant	Protein coding	KCNK17
							Noncoding transcript exon variant	Retained intron	KCNK17
							Regulatory region variant	CTCF binding site	
39311072	G	GCACACA	rs1373643967	7.4e-03	5.1e-03	6.7e-03	Intron variant	Protein coding	KCNK17
							Noncoding transcript exon variant	Retained intron	KCNK17
							Regulatory region variant	CTCF binding site	
39311072	G	GCACACACA	rs1373643967	4.7e-03	8.5e-03	5.8e-03	Intron variant	Protein coding	KCNK17
							Noncoding transcript exon variant	Retained intron	KCNK17
							Regulatory region variant	CTCF binding site	
39311072	GCA	G	rs764455277	3.1e-02	2.9e-02	3.1e-02	Intron variant	Protein coding	KCNK17
							Noncoding transcript exon variant	Retained intron	KCNK17
							Regulatory region variant	CTCF binding site	
39311072	GCACA	G	rs1384140897	2.8e-02	3.1e-02	2.9e-02	Intron variant	Protein coding	KCNK17
							Noncoding transcript exon variant	Retained intron	KCNK17

							Regulatory region variant	CTCF binding site	
39311072	GCACACA	G	rs200555541	1.3e-03	0	9.6e-04	Intron variant	Protein coding	KCNK17
							Noncoding transcript exon variant	Retained intron	KCNK17
							Regulatory region variant	CTCF binding site	
39311072	GCACACACA	G	rs749145766	6.7e-04	0	4.8e-04	Intron variant	Protein coding	KCNK17
							Noncoding transcript exon variant	Retained intron	KCNK17
							Regulatory region variant	CTCF binding site	
39311072	GCACACACACA	G	rs1425337022	3.4e-03	3.4e-03	3.4e-03	Intron variant	Protein coding	KCNK17
							Noncoding transcript exon variant	Retained intron	KCNK17
							Regulatory region variant	CTCF binding site	
39311072	GCACACACACACACACA	G	rs1448778094	1.1e-02	2.0e-02	1.4e-03	Intron variant	Protein coding	KCNK17
							Noncoding transcript exon variant	Retained intron	KCNK17
							Regulatory region variant	CTCF binding site	
39311072	GCACACACACACACACACA	G	rs72125468	6.7e-04	0	4.8e-04	Intron variant	Protein coding	KCNK17
							Noncoding transcript exon variant	Retained intron	KCNK17

							Regulatory region variant	CTCF binding site	
39311072	GCACACACACACACACACACA	G	rs1194978005	2.0e-03	3.4e-03	2.4e-03	Intron variant	Protein coding	KCNK17
							Noncoding transcript exon variant	Retained intron	KCNK17
							Regulatory region variant	CTCF binding site	
39311072	GCACACACACACACACACACACACACA	G	rs779801560	1.1e-02	3.4e-03	8.7e-03	Intron variant	Protein coding	KCNK17
							Noncoding transcript exon variant	Retained intron	KCNK17
							Regulatory region variant	CTCF binding site	
39311072	GCACACACACACACACACACACACACA	GCACACA	rs1194978005	6.7e-04	0	4.8e-04	Intron variant	Protein coding	KCNK17
							Noncoding transcript exon variant	Retained intron	KCNK17
							Regulatory region variant	CTCF binding site	
39311072	GCACACACACACACACACACACACACA	GCACACACA	rs72125468	2.7e-02	3.4e-03	2.0e-02	Intron variant	Protein coding	KCNK17
							Noncoding transcript exon variant	Retained intron	KCNK17
							Regulatory region variant	CTCF binding site	
39311072	GCACACACACACACACACACACACACA	GCACACACACA	rs1448778094	1.1e-02	1.7e-03	8.7e-03	Intron variant	Protein coding	KCNK17
							Noncoding transcript exon variant	Retained intron	KCNK17

							Regulatory region variant	CTCF binding site	
39311072	GCACACACACACACACACACACACACA	GCACACACACACACACA	rs1425337022	1.3e-03	1.7e-03	1.4e-03	Intron variant	Protein coding	KCNK17
							Noncoding transcript exon variant	Retained intron	KCNK17
							Regulatory region variant	CTCF binding site	
39311072	GCACACACACACACACACACACACACA	GCACACACACACACACACA	rs749145766	1.9e-02	1.0e-02	1.7e-02	Intron variant	Protein coding	KCNK17
							Noncoding transcript exon variant	Retained intron	KCNK17
							Regulatory region variant	CTCF binding site	
39311072	GCACACACACACACACACACACACACA	GCACACACACACACACACACACA	rs1384140897	1.5e-02	3.4e-03	1.2e-02	Intron variant	Protein coding	KCNK17
							Noncoding transcript exon variant	Retained intron	KCNK17
							Regulatory region variant	CTCF binding site	
39311072	GCACACACACACACACACACACACACA	GCACACACACACACACACACACACA	rs764455277	5.4e-03	8.5e-03	6.3e-03	Intron variant	Protein coding	KCNK17
							Noncoding transcript exon variant	Retained intron	KCNK17
							Regulatory region variant	CTCF binding site	
39311072	GCACACACACACACACACACACACACA	GCACACACACACACACACACACACACACA	rs1373643967	1.3e-03	0	9.6e-04	Intron variant	Protein coding	KCNK17

							Noncoding transcript exon variant	Retained intron	KCNK17
							Regulatory region variant	CTCF binding site	
39311072	GCACACACACACACACACACACACACA	GCACACACACACACACACACACACACACACA	rs1373643967	4.7e-03	1.7e-03	3.9e-03	Intron variant	Protein coding	KCNK17
							Noncoding transcript exon variant	Retained intron	KCNK17
							Regulatory region variant	CTCF binding site	
39311072	GCACACACACACACACACACACACACA	GCACACACACACACACACACACACACACACACA	rs1373643967	0	3.4e-03	9.6e-04	Intron variant	Protein coding	KCNK17
							Noncoding transcript exon variant	Retained intron	KCNK17
							Regulatory region variant	CTCF binding site	
39311072	GCACACACACACACACACACACACACA	GCACACACACACACACACACACACACACACACACA	rs1373643967	6.7e-04	0	4.8e-04	Intron variant	Protein coding	KCNK17
							Noncoding transcript exon variant	Retained intron	KCNK17
							Regulatory region variant	CTCF binding site	
39311109	C	T	rs2395723	2.0e-03	0	1.4e-03	Intron variant	Protein coding	KCNK17
							Noncoding transcript exon variant	Retained intron	KCNK17

							Regulatory region variant	CTCF binding site	
39311223	T	C	rs1544049	2.7e-03	0	1.9e-03	Intron variant	Protein coding	KCNK17
							Noncoding transcript exon variant	Retained intron	KCNK17
39311254	A	C		6.7e-04	0	4.8e-04	Intron variant	Protein coding	KCNK17
							Noncoding transcript exon variant	Retained intron	KCNK17
39311273	G	A	rs375681129	8.0e-03	1.7e-03	6.3e-03	Intron variant	Protein coding	KCNK17
							Noncoding transcript exon variant	Retained intron	KCNK17
39311355	G	A	rs1544050	2.7e-03	0	1.9e-03	Intron variant	Protein coding	KCNK17
							Noncoding transcript exon variant	Retained intron	KCNK17
39311360	G	A	rs151078165	6.7e-03	3.4e-03	5.8e-03	Intron variant	Protein coding	KCNK17
							Noncoding transcript exon variant	Retained intron	KCNK17
39311639	T	C	rs6907212	6.7e-04	1.7e-03	9.6e-04	Intron variant	Protein coding	KCNK17
							Noncoding transcript exon variant	Retained intron	KCNK17
39311654	T	A	rs6907218	6.7e-04	1.7e-03	9.6e-04	Intron variant	Protein coding	KCNK17
							Noncoding transcript exon variant	Retained intron	KCNK17
39311676	T	G	rs6907225	6.7e-04	1.7e-03	9.6e-04	Intron variant	Protein coding	KCNK17

							Noncoding transcript exon variant	Retained intron	KCNK17
39311754	T	C	rs6907394	6.7e-04	1.7e-03	9.6e-04	Intron variant	Protein coding	KCNK17
							Noncoding transcript exon variant	Retained intron	KCNK17
39311908	G	A	rs6928045	6.7e-04	1.7e-03	9.6e-04	Intron variant	Protein coding	KCNK17
							Noncoding transcript exon variant	Retained intron	KCNK17
39311927	C	G	rs6927737	6.7e-04	1.7e-03	9.6e-04	Intron variant	Protein coding	KCNK17
							Noncoding transcript exon variant	Retained intron	KCNK17
39311946	CACTGAGCA	C	rs137963971	3.4e-02	4.4e-02	3.7e-02	Intron variant	Protein coding	KCNK17
							Noncoding transcript exon variant	Retained intron	KCNK17
39312039	A	G	rs6927793	6.7e-04	1.7e-03	9.6e-04	Intron variant	Protein coding	KCNK17
							Noncoding transcript exon variant	Retained intron	KCNK17
39312055	G	A	rs551423178	1.3e-03	3.4e-03	1.9e-03	Intron variant	Protein coding	KCNK17
							Noncoding transcript exon variant	Retained intron	KCNK17
39312227	G	T	rs571188242	6.7e-04	0	4.8e-04	Intron variant	Protein coding	KCNK17
							Noncoding transcript exon variant	Retained intron	KCNK17
39312302	A	AAAG	rs144225973	6.7e-04	1.7e-03	9.6e-04	Intron variant	Protein coding	KCNK17

							Noncoding transcript exon variant	Retained intron	KCNK17
39312309	T	G	rs777636059	6.7e-04	0	4.8e-04	Intron variant	Protein coding	KCNK17
							Noncoding transcript exon variant	Retained intron	KCNK17
39312340	T	A	rs75113401	6.7e-04	1.7e-03	9.6e-04	Intron variant	Protein coding	KCNK17
							Noncoding transcript exon variant	Retained intron	KCNK17
39312470	G	A	rs75681028	6.7e-04	1.7e-03	9.6e-04	Intron variant	Protein coding	KCNK17
							Noncoding transcript exon variant	Retained intron	KCNK17
39312596	G	A	rs1302681257	0	1.7e-03	4.8e-04	Intron variant	Protein coding	KCNK17
							Noncoding transcript exon variant	Retained intron	KCNK17
39312621	C	T	rs141576163	1.4e-02	1.9e-02	1.5e-02	Intron variant	Protein coding	KCNK17
							Noncoding transcript exon variant	Retained intron	KCNK17
39312658	A	G	rs9462523	6.7e-04	1.7e-03	9.6e-04	Intron variant	Protein coding	KCNK17
							Noncoding transcript exon variant	Retained intron	KCNK17
39312665	C	T	rs9471061	6.7e-04	1.7e-03	9.6e-04	Intron variant	Protein coding	KCNK17
							Noncoding transcript exon variant	Retained intron	KCNK17
39312668	C	G	rs9471062	6.7e-04	1.7e-03	9.6e-04	Intron variant	Protein coding	KCNK17

							Noncoding transcript exon variant	Retained intron	KCNK17
39312673	G	A	rs187837093	4.0e-03	6.8e-03	4.8e-03	Intron variant	Protein coding	KCNK17
							Noncoding transcript exon variant	Retained intron	KCNK17
39312686	G	T	rs9462524	6.7e-04	1.7e-03	9.6e-04	Intron variant	Protein coding	KCNK17
							Noncoding transcript exon variant	Retained intron	KCNK17
39312687	A	G	rs9462525	6.7e-04	1.7e-03	9.6e-04	Intron variant	Protein coding	KCNK17
							Noncoding transcript exon variant	Retained intron	KCNK17
**PRE on chromosome 9 spanning position 116284900-116285300**									
**Variant position**	**Reference allele**	**Alternative allele**	**SNP identifier**	**MAF in cases**	**MAF in controls**	**Cohort MAF**	**Consequence**	**Biotype**	**Gene**
116285129	C	CTTTTTCT	rs201987126	2.3e-02	2.4e-02	2.3e-02	Intron variant	Protein coding	PAPPA
116285142	C	CTTTCTT	rs753008319	3.4e-03	5.1e-03	3.9e-03	Intron variant	Protein coding	PAPPA
116285159	T	TTTTC	rs1268299746	6.7e-04	0	4.8e-04	Intron variant	Protein coding	PAPPA
116285167	C	T	rs370162234	6.7e-04	0	4.8e-04	Intron variant	Protein coding	PAPPA
116285171	C	CT	rs1186068742	6.7e-04	5.1e-03	1.9e-03	Intron variant	Protein coding	PAPPA
116285171	C	CTT	rs1186068742	2.7e-03	0	1.9e-03	Intron variant	Protein coding	PAPPA
116285171	C	CTTT	rs1186068742	8.7e-03	2.4e-02	1.3e-02	Intron variant	Protein coding	PAPPA
116285171	C	CTTTT	rs1186068742	1.1e-02	1.2e-02	1.1e-02	Intron variant	Protein coding	PAPPA

116285171	C	CTTTTT	rs1186068742	1.5e-02	2.2e-02	1.7e-02	Intron variant	Protein coding	PAPPA
116285171	C	CTTTTTT	rs1186068742	0	3.4e-03	9.6e-04	Intron variant	Protein coding	PAPPA
116285171	C	CTTTTTTT	rs1186068742	4.7e-03	5.1e-03	4.8e-03	Intron variant	Protein coding	PAPPA
116285171	C	CTTTTTTTTTTTTTTT		6.7e-04	0	4.8e-04	Intron variant	Protein coding	PAPPA
116285171	C	CTTTTTTTTTTTTTTTTTTT		6.7e-04	0	4.8e-04	Intron variant	Protein coding	PAPPA
116285171	C	CTTTTTTTTTTTTTTTTTTTTTTTTT		0	1.7e-03	4.8e-04	Intron variant	Protein coding	PAPPA
116285171	C	T	rs373357579	0	1.7e-03	4.8e-04	Intron variant	Protein coding	PAPPA
116285171	CT	C	rs1461822674	2.0e-03	3.4e-03	2.4e-03	Intron variant	Protein coding	PAPPA
116285171	CT	CTT	rs1186068742	7.4e-03	1.5e-02	9.6e-03	Intron variant	Protein coding	PAPPA
116285171	CT	CTTTT	rs1186068742	0	3.4e-03	9.6e-04	Intron variant	Protein coding	PAPPA
116285171	CT	CTTTTTT	rs1186068742	8.7e-03	0	6.3e-03	Intron variant	Protein coding	PAPPA
116285171	CT	CTTTTTTT	rs1186068742	1.6e-02	1.5e-02	1.6e-02	Intron variant	Protein coding	PAPPA
116285171	CT	CTTTTTTTT	rs1186068742	1.3e-03	3.4e-03	1.9e-03	Intron variant	Protein coding	PAPPA
116285176	T	C	rs866553011	3.4e-03	6.8e-03	4.3e-03	Intron variant	Protein coding	PAPPA
116285199	C	T	rs752534102	6.7e-04	0	4.8e-04	Intron variant	Protein coding	PAPPA
116285190	TGACAGA	T	rs1269494360	6.7e-04	0	4.8e-04	Intron variant	Protein coding	PAPPA
116285207	G	T	rs1379529994	6.7e-04	0	4.8e-04	Intron variant	Protein coding	PAPPA
							Regulatory region variant	CTCF binding site	
116285200	TCAC	T		6.7e-04	0	4.8e-04	Intron variant	Protein coding	PAPPA

							Regulatory region variant	CTCF binding site	
**PRE on chromosome 17 spanning position 79288362-81288362**									
**Variant position**	**Reference allele**	**Alternative allele**	**SNP identifier**	**MAF in cases**	**MAF in controls**	**Cohort MAF**	**Consequence**	**Biotype**	**Gene**
79743000	G	A	rs151138055	1.9e-02	1.7e-02	1.9e-02	Intergenic variant		
							Regulatory region variant	enhancer	
							TFBS variant		
79743526	C	T	rs186983861	2.7e-03	3.4e-03	2.9e-03	Intergenic variant		
79743695	C	T	rs782085970	0	3.4e-03	9.6e-04	Intergenic variant		
79743782	G	A	rs1022499002	0	3.4e-03	9.6e-04	Intergenic variant		
79743905	C	G	rs142093299	4.0e-03	1.7e-03	3.4e-03	Intergenic variant		
79744165	G	A	rs574355800	2.0e-03	1.7e-03	1.9e-03	Intergenic variant		
79744425	A	G	rs543023666	4.0e-03	5.1e-03	4.3e-03	Intergenic variant		
79744508	T	C	rs931838327	0	3.4e-03	9.6e-04	Intergenic variant		
79744530	T	C	rs7350911	2.7e-03	0	1.9e-03	Intergenic variant		
79744569	G	A	rs559729244	1.3e-03	1.7e-03	1.4e-03	Intergenic variant		
79744691	T	C	rs6565482	6.7e-04	0	4.8e-04	Intergenic variant		
79744748	C	T	rs139975055	8.0e-03	1.9e-02	1.1e-02	Intergenic variant		
79744847	T	G		6.7e-04	1.7e-03	9.6e-04	Intergenic variant		
79744911	T	C	rs1472495903	6.7e-04	0	4.8e-04	Intergenic variant		

79744913	T	C		6.7e-04	0	4.8e-04	Intergenic variant		
79745096	C	T		6.7e-04	0	4.8e-04	Intergenic variant		
79745214	G	A	rs188506701	0	1.7e-03	4.8e-04	Intergenic variant		
79745401	C	T	rs77272891	4.8e-02	4.4e-02	4.7e-02	Intergenic variant		
79745660	G	A	rs4890023	6.7e-04	0	4.8e-04	Intergenic variant		
							Regulatory region variant	CTCF binding site	
79745744	CT	C		2.7e-03	0	1.9e-03	Intergenic variant		
							Regulatory region variant	CTCF binding site	
79746183	C	T	rs534833318	1.3e-02	1.0e-02	1.3e-02	Intergenic variant		
							Regulatory region variant	CTCF binding site	
79746195	T	TAAAAA		1.3e-03	0	9.6e-04	Intergenic variant		
							Regulatory region variant	CTCF binding site	
79746195	T	TAAAAAAAAA		0	3.4e-03	9.6e-04	Intergenic variant		
							Regulatory region variant	CTCF binding site	
79746195	TAAAAAAAAAAA	T	rs1374326859	3.4e-03	1.7e-03	2.9e-03	Intergenic variant		
							Regulatory region variant	CTCF binding site	
79746195	TAAAAAAAAAAAA	T	rs1302548755	2.3e-02	1.2e-02	2.0e-02	Intergenic variant		

							Regulatory region variant	CTCF binding site	
79746195	TAAAAAAAAAAAAA	T	rs1413301088	3.5e-02	6.1e-02	4.2e-02	Intergenic variant		
							Regulatory region variant	CTCF binding site	
79746195	TAAAAAAAAAAAAAA	T	rs1377694494	3.5e-02	3.6e-02	3.5e-02	Intergenic variant		
							Regulatory region variant	CTCF binding site	
79746195	TAAAAAAAAAAAAAAA	T	rs1174695423	1.0e-02	6.8e-03	9.1e-03	Intergenic variant		
							Regulatory region variant	CTCF binding site	
79746195	TAAAAAAAAAAAAAAAAA	T	rs1362583674	7.4e-03	1.5e-02	9.6e-03	Intergenic variant		
							Regulatory region variant	CTCF binding site	
79746195	TAAAAAAAAAAAAAAAAAA	T	rs1156306872	2.0e-03	5.1e-03	2.9e-03	Intergenic variant		
							Regulatory region variant	CTCF binding site	
79746195	TAAAAAAAAAAAAAAAAAAA	T	rs1420415854	2.0e-03	5.1e-03	2.9e-03	Intergenic variant		
							Regulatory region variant	CTCF binding site	
79746195	TAAAAAAAAAAAAAAAAAAAA	T	rs1250765018	6.0e-03	8.5e-03	6.7e-03	Intergenic variant		
							Regulatory region variant	CTCF binding site	
79746195	TAAAAAAAAAAAAAAAAAAAAA	T	rs1186829406	2.0e-03	0	1.4e-03	Intergenic variant		

							Regulatory region variant	CTCF binding site	
79746195	TAAAAAAAAAAAAAAAAAAAAAAA	T	rs1259292043	1.7e-02	1.0e-02	1.5e-02	Intergenic variant		
							Regulatory region variant	CTCF binding site	
79746195	TAAAAAAAAAAAAAAAAAAAAAAA	TAAA	rs1250765018	6.7e-04	1.7e-03	9.6e-04	Intergenic variant		
							Regulatory region variant	CTCF binding site	
79746195	TAAAAAAAAAAAAAAAAAAAAAAA	TAAAAA	rs1156306872	8.7e-03	1.7e-03	6.7e-03	Intergenic variant		
							Regulatory region variant	CTCF binding site	
79746195	TAAAAAAAAAAAAAAAAAAAAAAA	TAAAAAA	rs1362583674	6.0e-03	1.7e-03	4.8e-03	Intergenic variant		
							Regulatory region variant	CTCF binding site	
79746195	TAAAAAAAAAAAAAAAAAAAAAAA	TAAAAAAA	rs1419578586	6.7e-04	0	4.8e-04	Intergenic variant		
							Regulatory region variant	CTCF binding site	
79746195	TAAAAAAAAAAAAAAAAAAAAAAA	TAAAAAAAA	rs1174695423	1.3e-03	8.5e-03	3.4e-03	Intergenic variant		
							Regulatory region variant	CTCF binding site	
79746195	TAAAAAAAAAAAAAAAAAAAAAAA	TAAAAAAAAA	rs1377694494	5.4e-03	5.1e-03	5.3e-03	Intergenic variant		
							Regulatory region variant	CTCF binding site	
79746195	TAAAAAAAAAAAAAAAAAAAAAAA	TAAAAAAAAAA	rs1413301088	1.9e-02	8.5e-03	1.6e-02	Intergenic variant		

							Regulatory region variant	CTCF binding site	
79746195	TAAAAAAAAAAAAAAAAAAAAAAA	TAAAAAAAAAAA	rs1302548755	3.4e-03	0	2.4e-03	Intergenic variant		
							Regulatory region variant	CTCF binding site	
79746195	TAAAAAAAAAAAAAAAAAAAAAAA	TAAAAAAAAAAAA	rs1374326859	6.7e-04	0	4.8e-04	Intergenic variant		
							Regulatory region variant	CTCF binding site	
79746195	TAAAAAAAAAAAAAAAAAAAAAAAA	T	rs1211346023	2.0e-03	3.4e-03	2.4e-03	Intergenic variant		
							Regulatory region variant	CTCF binding site	
79746196	A	C		6.7e-04	0	4.8e-04	Intergenic variant		
							Regulatory region variant	CTCF binding site	
