# Supplementary material for: Familial analysis reveals rare risk variants for migraine in regulatory regions
Source: Neurogenetics. 2020 Feb 19;21(3):149–57. doi: 10.1007/s10048-020-00606-5 (PMC7283211; doi:10.1007/s10048-020-00606-5)
Supplement: Supplementary file 6 — (PDF 28 kb) [file 10048_2020_606_MOESM5_ESM.pdf]

**Supplementary table 4.** ***Cis*****-eQTLs found for the rare variants segregating with migraine in the four regulatory regions.** The name of the migraine risk loci and the genomic positions of the regulatory regions is displayed. The SNP identifiers of the rare variants were found using the dbSNP build 152. For each rare variant with a significant *cis*-eQTL, the eQTL index SNP, eQTL gene, GTEx tissue, eQTL *p*-value is presented.

| Locus | Position (chromosome:start:end) | eQTL index SNP | eQTL gene | GTEx tissue | eQTL *p*-value |
|---|---|---|---|---|---|
| *PHACTR1* | CpG island (chr6:13486092:13488560) | rs62385806 | *GFOD1* | Skin; Sun Exposed (Lower leg) | 1.6e-4 |
| | | rs982391 | *GFOD1* | Skin; Sun Exposed (Lower leg) | 8.4e-5 |
| *KCNK5* | PRE (chr6:39310446:39312846) | rs72855550 | *KCNK17* | Esophagus; Muscularis | 5.0e-6 |
| | | | | Artery; Tibial | 1.3e-4 |
| | | rs1544049 | *KCNK17* | Whole Blood | 2.5e-18 |
| | | | | Pancreas | 6.0e-11 |
| | | | | Esophagus; Mucosa | 2.8e-10 |
| | | | | Prostate | 2.3e-9 |
| | | | | Esophagus; Muscularis | 9.5e-9 |
| | | | | Esophagus; Gastroesophageal Junction | 6.6e-8 |
| | | | | Spleen | 4.2e-7 |
| | | | | Minor Salivary Gland | 4.4e-7 |
| | | | | Small Intestine; Terminal Ileum | 5.7e-6 |
| | | | | Artery; Tibial | 1.2e-5 |
| | | | | Adipose; Subcutaneous | 3.3e-5 |
| | | | | Whole Blood | 8.9e-17 |

| | | | | | |
|---|---|---|---|---|---|
| | | | | Pancreas | 9.3e-12 |
| | | | | Esophagus; Mucosa | 3.7e-10 |
| | | | | Prostate | 9.7e-10 |
| | | | | Esophagus; Muscularis | 7.7e-8 |
| | | | | Minor Salivary Gland | 9.5e-8 |
| | | | | Esophagus; Gastroesophageal Junction | 2.8e-7 |
| | | | | Artery; Tibial | 3.9e-7 |
| | | | | Spleen | 2.6e-6 |
| | | | | Testis | 2.9e-5 |
| | | rs1544050 | *KIF6* | Artery; Aorta | 1.1e-4 |
| *RNF213* | PRE (chr17:79742606:79746206) | rs151138055 | *CBX2* | Cells; Cultured fibroblasts | 2.6e-5 |
| | | rs7350911 | *LINC02078* | Spleen | 1.1e-6 |
| | | rs7350911 | *ENPP7* | Lung | 2.2e-5 |
| | | rs6565482 | *TBC1D16* | Breast; Mammary Tissue | 2.4e-6 |
| | | rs6565482 | *CARD14* | Skin; Not Sun Exposed (Suprapubic) | 5.1e-6 |
